# Antiviral RNAi in Insects and Mammals: Parallels and Differences

**DOI:** 10.3390/v11050448

**Published:** 2019-05-16

**Authors:** Susan Schuster, Pascal Miesen, Ronald P. van Rij

**Affiliations:** Department of Medical Microbiology, Radboud University Medical Center, Radboud Institute for Molecular Life Sciences, 6500 HB Nijmegen, The Netherlands; susan.schuster@radboudumc.nl (S.S.); pascal.miesen@radboudumc.nl (P.M.)

**Keywords:** small interfering RNA, RNA interference, RNA virus, antiviral defense, innate immunity, interferon, stem cells

## Abstract

The RNA interference (RNAi) pathway is a potent antiviral defense mechanism in plants and invertebrates, in response to which viruses evolved suppressors of RNAi. In mammals, the first line of defense is mediated by the type I interferon system (IFN); however, the degree to which RNAi contributes to antiviral defense is still not completely understood. Recent work suggests that antiviral RNAi is active in undifferentiated stem cells and that antiviral RNAi can be uncovered in differentiated cells in which the IFN system is inactive or in infections with viruses lacking putative viral suppressors of RNAi. In this review, we describe the mechanism of RNAi and its antiviral functions in insects and mammals. We draw parallels and highlight differences between (antiviral) RNAi in these classes of animals and discuss open questions for future research.

## 1. Introduction 

RNA interference (RNAi) or RNA silencing was first described in the model organism *Caenorhabditis elegans* [1] and following this ground-breaking discovery, studies in the field of small, noncoding RNAs have advanced tremendously. RNAi acts, with variations, in all eukaryotes ranging from unicellular organisms to complex species from the plant and animal kingdoms [2]. The key concept of all RNA silencing pathways is the association of single-stranded small RNAs of 20–30 nucleotides (nt) to a protein of the Argonaute superfamily [3,4]. In animals, three classes of small RNAs exist: small interfering RNAs (siRNAs), microRNAs (miRNAs) and PIWI-interacting RNAs (piRNAs) [2,5]. These RNAs guide Argonaute proteins onto target RNAs via Watson-Crick base pairing, usually resulting in gene silencing [6]. Whereas all three pathways adhere to the general concept of RNA silencing pathways, they differ in the mechanism for small RNA biogenesis and effector functions. For example, biogenesis of siRNAs and miRNAs depends on processing of double-stranded RNA (dsRNA) precursors into small RNAs by RNase-III Dicer enzymes [6], whereas piRNA biogenesis is Dicer independent. 

Early on, it was recognized that RNAi could be a mechanism for antiviral defense, and, in fact, siRNAs were first detected in virus-infected plants [7,8,9]. It is now well established that RNAi is a major defense mechanism against parasitic nucleic acids in diverse organisms, including fungi, plants, and invertebrates [10,11,12]. Thus, recognition and processing of viral dsRNA into viral siRNAs (vsiRNAs) initiates a potent antiviral RNAi response that restricts virus accumulation. However, even though the mechanism of RNAi is evolutionarily conserved in mammals, the degree to which it contributes to antiviral defense has been a matter of debate. Positive and negative-sense RNA viruses were recently proposed to be a substrate for the RNAi pathway in several mammalian cell culture and animal models [13,14,15], yet conflicting evidence has also emerged in several studies that failed to detect vsiRNAs [16,17,18,19]. In vertebrates, RNAi coincides with the dsRNA-activated protein-based interferon response and recent findings suggest that mammalian RNAi is inhibited by the interferon response, suggestive of competition between both pathways [20,21]. 

In this review, we will discuss recent work on the antiviral function of RNAi in mammals, focusing on negative and positive-sense RNA viruses (excluding retroviruses). We will first describe the principal concepts of RNAi in insects and mammals (for a review on RNA silencing in plants, see [10]) and briefly discuss interferon-based antiviral immunity in mammals. Finally, we will discuss the antiviral activity of RNAi in insects and different mammalian experimental systems. Special attention will be given to stem cells, which seem to have specific characteristics, both in the interferon response and antiviral RNAi. To avoid ambiguity, we will only consider “classical” antiviral RNAi, in which viral dsRNA is processed into viral siRNAs to limit virus infection; we will not consider miRNA-dependent effects on virus replication. 

## 2. The Mechanism of RNAi

Although RNA silencing pathways adhere to the same general concepts, paralogs of Dicer and Argonaute genes have emerged via duplications during eukaryotic evolution. This, along with the recruitment of different accessory proteins and co-factors, has led to functional diversification or specialization in different organisms [22]. For example, insects such as the fruit fly *Drosophila melanogaster* encode two Dicer genes, of which Dicer-1 mediates miRNA biogenesis, whereas Dicer-2 is responsible for siRNA biogenesis [6]. In contrast, mammals only encode a single Dicer that generates both miRNAs and siRNAs. Likewise, Argonaute-2 is responsible for siRNA-mediated target RNA cleavage in insects, whereas Argonaute-1 mediates miRNA-dependent gene silencing. Mammals, in contrast, encode four Argonaute genes, all of which engage in microRNA-guided gene silencing, and only Argonaute-2 is capable of cleaving target RNAs (also referred to as slicing) to mediate siRNA-dependent RNAi.

Below, we will discuss the siRNA and miRNA pathways of insects and mammals in more detail. Although the piRNA pathway has been suggested to mediate antiviral defense, especially in vector mosquitoes [23], piRNAs have not been studied in the context of viral infection in mammals and will not be discussed.

### 2.1. The siRNA Pathway in Insects

**Figure 1:**
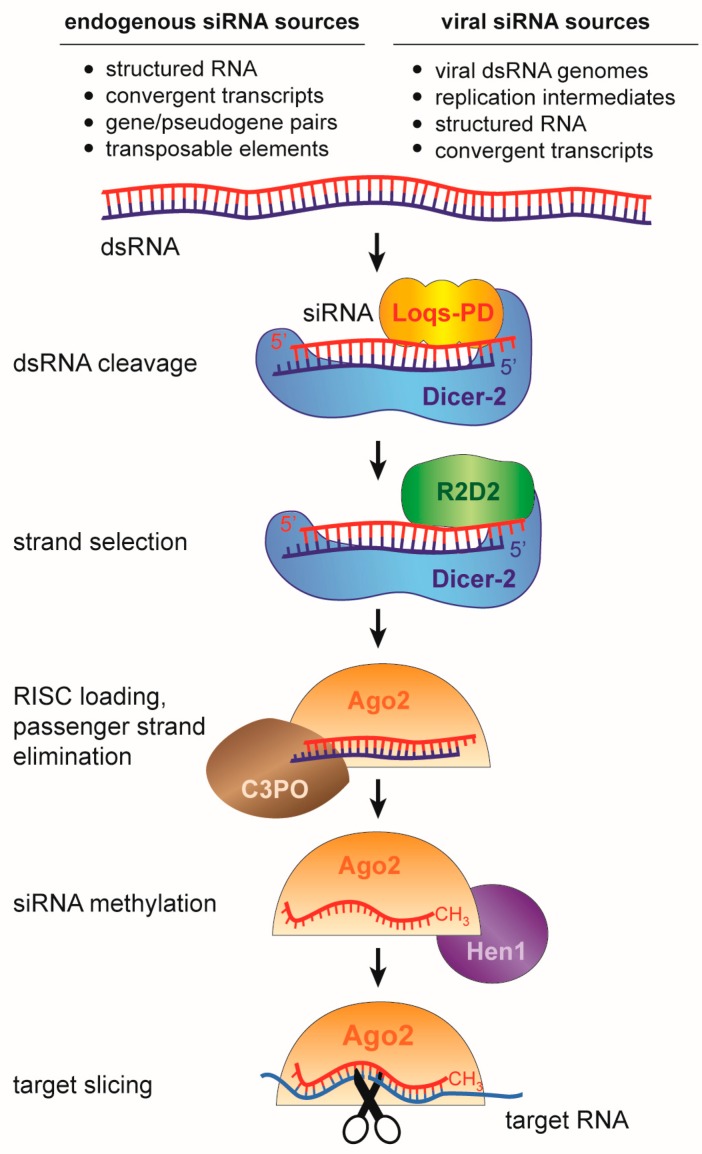
The small interfering RNA (siRNA) pathway in *Drosophila melanogaster*. Double-stranded RNA precursors of different sources are processed by Dicer-2 into short interfering RNAs of ~21 nt in size. The siRNA duplex is loaded into an Argonaute2 containing RISC complex, where one strand (passenger) is degraded, and the guide strand is retained. The guide strand mediates target RNA recognition through Watson-Crick base pairing, followed by target cleavage (slicing) by Argonaute. Loqs-PD is required for endo-siRNA biogenesis, but dispensable for viral siRNA (vsiRNA) biogenesis.

**Figure 2:**
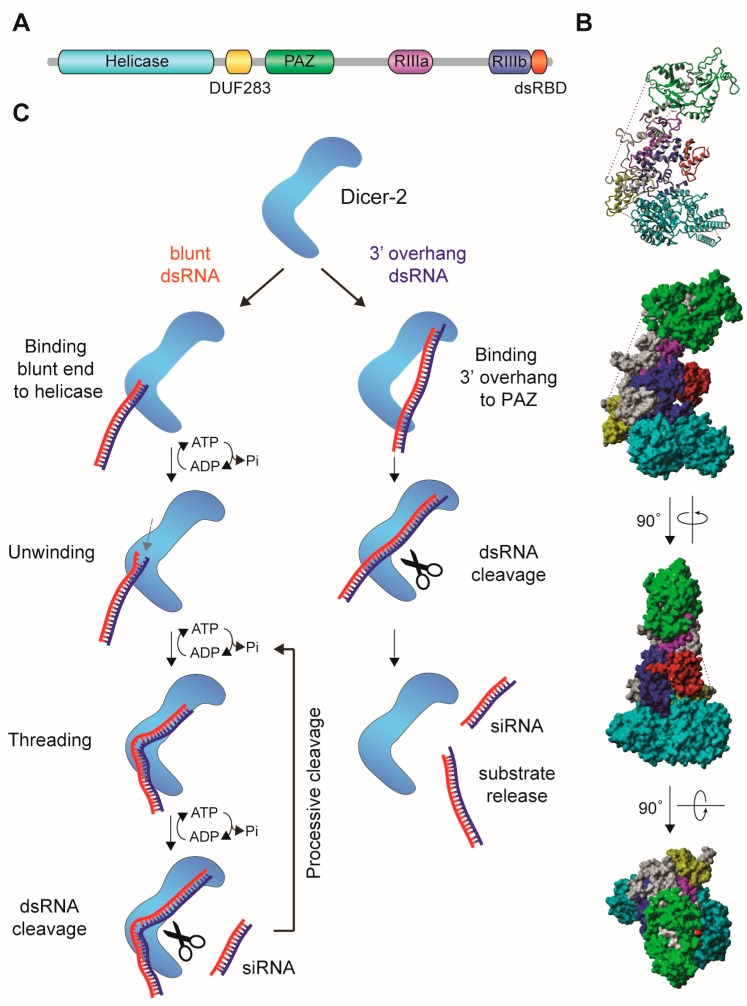
Dicer proteins process double-stranded RNA (dsRNA) into small interfering RNA (siRNA). (**A**) Schematic representation of the domain organization of human Dicer protein [40]. RIIIa, RNase-IIIa; RIIIb, RNase-IIIb (**B**) Cryo-EM structure of human Dicer. Protein domains are colored in accordance to the scheme in A. The structure was determined by Liu et al. [41], and the published PDB file (5ZAM) was edited in Yasara View [42]. *Drosophila* Dicer-2 has a similar domain structure and L-shaped Cryo-EM structure as human Dicer [40]. (**C**) Schematic representation of the recognition and cleavage of dsRNA with a 3’ overhang and dsRNA with blunt termini by *Drosophila* Dicer-2, proposed by Sinha and colleagues [40]. Substrates with a 3’ overhang were proposed to bind the PAZ-Platform domains (referred to as PAZ in panel A) via the 3’ terminal overhang. Blunt-ended termini bind to the helicase domain and the dsRNA threads through this domain, after which cleavage occurs by the two RNaseIII domains. The latter mode results in processive, ATP-dependent cleavage of dsRNA and may contribute to efficient production of vsiRNAs for antiviral defense.

The “classical” RNAi mechanism, uncovered by Fire and Mello [1], is triggered by the presence of double-stranded RNA (dsRNA) in the cytoplasm. This initiates a series of processing steps that eventually results in the production of siRNAs that associate with an Argonaute protein (Figure 1). In insects, the RNase-III enzyme Dicer-2 recognizes cytoplasmic dsRNA and cleaves it into 21 nt siRNA duplexes with characteristic two-nucleotide overhangs at the 3’ ends of both strands (Figure 2) [24,25,26,27]. One of the two strands (the guide strand) is selectively incorporated into the RNA-induced silencing complex (RISC) with at its catalytic core the Argonaute-2 (Ago2) protein. The complementary strand (the passenger strand) is degraded in a process that requires Ago2 and the endonuclease Component 3 Promoter of RISC (C3PO) [28,29,30,31]. Selection of the guide and passenger strand is a non-stochastic process and involves the activity of the Dicer-2-associated co-factor R2D2 [32,33]. R2D2 probes the thermodynamic stability of the siRNA duplex and binds the more stable 5’ end, eventually defining the passenger strand. Dicer-2 selects the opposite strand that will be loaded as guide strand into Ago2 [34]. Dicer-2 processing and RISC loading is further promoted by the activity of co-factors including the dsRNA binding protein Loquacious (PD isoform, Loqs-PD), Ars2 and heat shock proteins [35,36,37,38]. These proteins enhance siRNA biogenesis by stabilizing the RNA-protein complexes or facilitating conformational changes during RISC loading. After the guide strand is stably bound by Ago2, it is 2’-*O*-methylated at the 3’ terminal nucleotide by the RNA methyl-transferase DmHen1 finalizing the maturation of an siRNA-loaded RISC [39].

**Figure 3:**
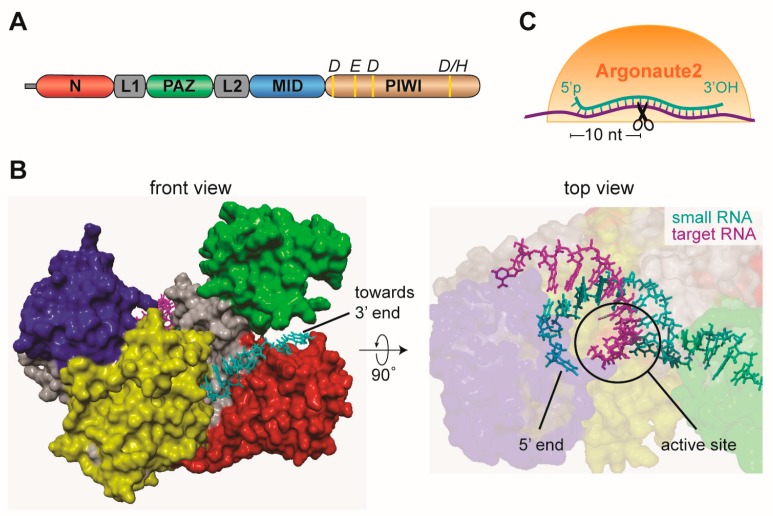
Argonaute proteins are at the core of small RNA silencing pathways. (**A**) Schematic representation of the domain organization of mammalian Argonaute and the conserved residues required for slicer activity. (**B**) Crystal structure of human AGO2 in association with a guide RNA and a target RNA base pairing from nucleotide 2 to 8. Protein domains are colored in accordance to the scheme in A. The structure was determined by Schirle and colleagues [49] and the published PDB file (4W5Q) was edited in Yasara View. (**C**) Schematic representation of target slicing by Argonaute proteins.

Two models of substrate processing depending on their termini have been proposed for Dicer-2 [40]. Substrates with 3’ overhangs are cleaved distributively by Dicer-2 in an ATP independent manner, releasing the dsRNA substrate after each cleavage. In contrast, dsRNA with blunt termini are locally unwound, with one of the strand threading through the helicase domain in an ATP dependent manner, after which the dsRNA re-anneals and becomes processively cleaved [40] (Figure 2C). Ago2-bound siRNAs recognize target RNAs via Watson-Crick base pairing and usually complementarity across the entire length of the siRNA/target duplex is required for efficient target cleavage. An exception is the first nucleotide of the siRNA, which is locked in a pocket of the Ago2 MID/PIWI domain [43] (Figure 3A,B). Upon formation of the siRNA/target RNA duplex, Ago2 cleaves the target RNA between nucleotide ten and eleven counted from the 5’ end of the siRNA (slicing, Figure 3C) [25,26,28,44]. This small RNA-mediated endonuclease activity (slicing) requires the catalytic DEDX tetrad (where X is D or H) in the PIWI domain of Argonaute proteins (Figure 3A) [45,46]. This motif is conserved amongst slicing-competent Argonaute proteins; nonetheless it is not sufficient for slicing activity since some slicing-incompetent Argonaute proteins also contain the motif [47]. After cleavage of target RNA, the slicing products are quickly degraded by cellular ribonucleases [48].

Endogenous sources of dsRNA are long inverted repeats that fold into perfectly complementary hairpins or transcripts that are derived from convergent transcription. In addition, gene-pseudogene pairs and transposon insertions are potential sources of dsRNA when they express transcripts with full or partial complementarity (Figure 1). These genome-encoded dsRNA molecules are processed into endogenous siRNAs (endo-siRNA) that have been implicated in transposon control and anecdotally in the regulation of gene expression [50,51,52,53,54]. Yet, dsRNA is usually not very abundant in healthy, uninfected cells and the major function of this pathway seems to be defense against foreign dsRNA of viral origin [55] (discussed in Section 4). 

### 2.2. The miRNA Pathway in Insects

miRNAs are an endogenous class of small RNAs, expressed by plants, animals, protists and even viruses [2]. Biogenesis of animal miRNAs resembles siRNA biogenesis, with some differences including the origin of precursor RNAs. miRNAs are processed from genome-encoded hairpins, called primary-miRNAs (pri-miRNAs) that are transcribed by RNA polymerase II and, less frequently, by RNA polymerase III [56,57,58]. Pri-miRNAs are typically a few kb in length [59] and harbor either single or multiple local stem-loop structures that undergo a series of maturation steps to generate an Argonaute-associated miRNA [60]. Typically, these stem-loops are ~80 nt in size and consist of two imperfectly base pairing arms, separated by a single-stranded loop region [61]. They are released from the pri-miRNA transcript in the nucleus by the microprocessor complex, consisting of the RNase-III enzyme Drosha and its co-factor Pasha [62,63,64,65,66]. Endonucleolytic cleavage by Drosha near the base of the hairpin produces the precursor miRNA (pre-miRNA), a ~60 nt small RNA hairpin with a two-nucleotide overhang at the 3’ end, indicative of RNase-III processing [66]. Subsequently, the pre-miRNA is exported from the nucleus via the Ran-GTP dependent nuclear exporter Exportin-5 [67,68,69,70]. In the cytoplasm, another RNase-III enzyme, Dicer-1, in a complex with the PB isoform of Loqs cleaves off the loop of the pre-miRNA resulting in an RNA heteroduplex with two-nucleotide overhangs at both 3’ ends [24,71,72]. One of the two strands is selectively incorporated into the Argonaute-1 containing miRNA induced silencing complex (miRISC) [73,74]. Strand selection is thought to be primarily based on the thermodynamic properties of the heteroduplex; usually the strand with the weaker stability at its 5’ end is incorporated into Ago1 [75,76]. The miRNA guides miRISC to target sites in the 3’ untranslated regions (UTR) of mRNAs, akin to target recognition in mammals [77] (described in Section 2.3).

### 2.3. RNAi Pathway in Mammals

**Figure 4:**
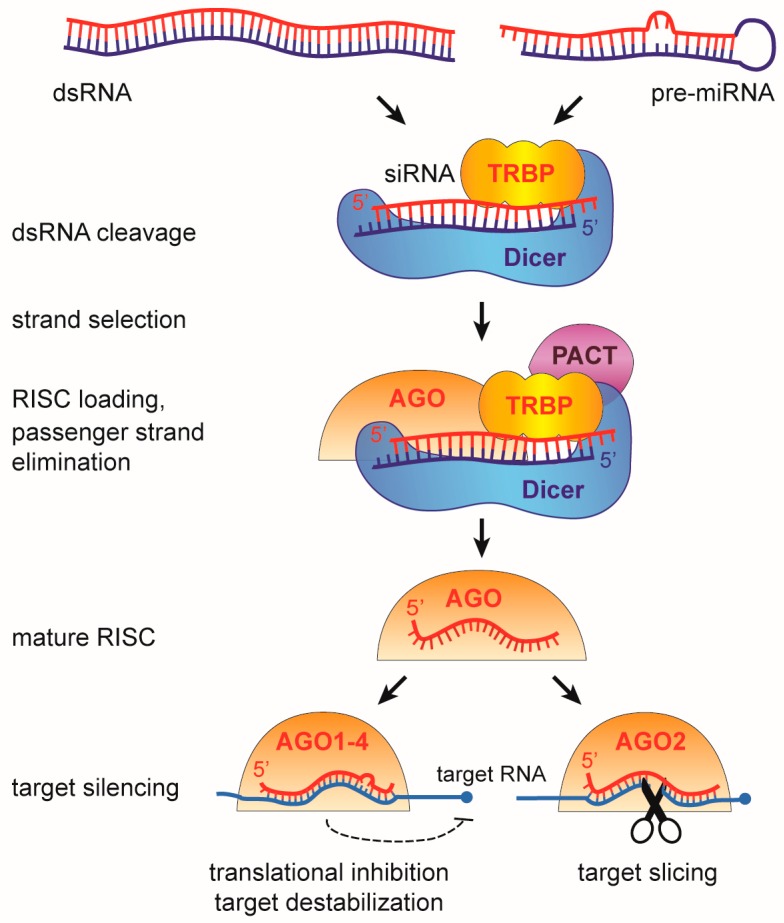
The RNA interference (RNAi) pathway in mammals. A single Dicer protein processes long dsRNA into siRNAs and pre-miRNAs into miRNA duplexes. These small RNAs are loaded into an Argonaute containing RISC complex, from which one of the strands is eliminated and degraded. The other strand, referred to as guide strand (for siRNAs) or the mature miRNA (for miRNAs), is retained and used to guide Argonaute onto target RNAs, resulting in cleavage (siRNA) or translational inhibition or target RNA destabilization (miRNA). The scheme shows the cytoplasmic stage of the miRNA pathway; the nuclear stage (pri-miRNA transcription, processing, and pre-miRNA nuclear export) is not shown.

Whereas the miRNA and siRNA pathways are largely independent in insects, siRNA and miRNA biogenesis and function in mammals depend on shared components (Figure 4), which results in crosstalk between these pathways. Like in insects, miRNAs in mammals are an abundant class of small RNAs of 21–22 nt in length [78] that are primarily produced from RNA polymerase II synthesized pri-miRNAs. These pri-miRNAs are processed into pre-miRNAs (pre-miRNAs) by the Microprocessor complex, consisting of the RNaseIII Drosha along with DGCR8 (DiGeorge Syndrome Critical Region 8) [79]. Pre-miRNAs are transported to the cytoplasm, where they are cleaved by Dicer into miRNA duplexes. These duplexes are loaded by Dicer and its co-factors TRBP (TAR RNA binding protein) and PACT (Protein kinase RNA activator) into an Argonaute (AGO) containing RISC complex, from which the passenger strand is eliminated. The RISC-associated mature miRNA base pairs with cognate messenger RNAs (mRNA), resulting in destabilization of target mRNAs or blocking their translation [24,61,80,81]. All four ubiquitously expressed mammalian AGO proteins mediate miRNA-mRNA interactions with approximately equivalent affinities [82,83,84] and overexpression experiments indicate that their miRNA binding patterns are similar [85,86]. 

In contrast to canonical Dicer-dependent miRNAs, non-canonical miRNAs bypass processing by Dicer or the Microprocessor complex. These non-canonical miRNAs can be derived from introns, small nucleolar RNAs (snoRNAs), and tRNAs [87,88,89,90,91,92,93]. For example, the mirtron pathway, which is also found in *D*. *melanogaster* and *C*. *elegans*, produces pre-miRNAs by the processing of introns by spliceosomes and debranching enzymes in the nucleus [94]. Another non-canonical miRNA is produced by processing of snoRNA ACA45 in a Drosha/DGCR8 independent, but Dicer dependent manner [88].

The miRISC complex is guided by the miRNA to target sites typically located in the 3’ UTRs of mRNAs [77]. Target recognition is initiated by a short nucleotide stretch at the 5’ end of the miRNA (position 2–8), the so-called seed sequence, accompanied with various degree of base pairing at the 3’ end [77,95,96,97]. Mechanisms for miRNA-mediated gene silencing include translational repression, de-adenylation, and enhancement of mRNA decay [77,98,99]. The majority of mRNAs is estimated to be regulated by miRNAs [100], and post-transcriptional regulation by miRNAs is thus implicated in almost all cell biological processes.

Although miRNA-mediated gene regulation seems to be the dominant function of mammalian RNAi, early evidence has already indicated that the siRNA pathway is functional in mammals. Transfection of synthetic siRNAs or expression of short-hairpin RNAs (shRNAs) with complementarity to a gene of interest was found to induce robust and sequence-specific RNAi, without activation of the interferon response as siRNAs are too short to be detected by dsRNA sensors (discussed in Section 3.1) [25,101]. Moreover, long dsRNA was reported to be functional in gene knockdown in embryonal teratocarcinoma cell lines that are interferon defective [102,103,104]. 

RNAi in mammals is characterized by processing of dsRNA by Dicer into 21–23 nt short interfering RNAs (siRNAs) [105]. Subsequently, siRNAs are preferentially loaded onto AGO1 or AGO2, of which only AGO2 possesses slicing activity in mammals [83,86]. After elimination of the passenger strand, the guide strand directs AGO2 onto complementary mRNA through base pairing. In contrast to the seed-based target recognition of miRNAs, siRNA targeting requires base pairing of the entire small RNA, resulting in target RNA cleavage by AGO2. As in insects, target cleavage occurs between nucleotide ten and eleven, counted from the 5’ end of the siRNA [106]. 

The evolutionary conservation of AGO2-mediated target cleavage in mammals suggests important functions for this activity. AGO2 efficiently mediates target repression independent of its slicer activity, as miRNA-mediated gene silencing in *AGO1*, *AGO3*, and *AGO4* deficient embryonic stem cells was comparable to control cells [83]. Yet, biogenesis of the non-canonical miRNA miR-451, implicated in the regulation of erythroid development, is Dicer-independent and instead depends on AGO2 catalysis [107]. In this case, the short length of the stem of only 17 bp likely explains why miR-451 fails to be processed by Dicer [108]. Besides being indispensable for miR-451 biogenesis, inactivation of *AGO2* by insertional mutagenesis in mice results in a lethal phenotype as only wild-type and heterozygous offspring are observed [86]. In addition, loss of AGO2 results in a severe developmental phenotype, including a defect or failure in neural tube closure and mispatterning of brain structures [86]. The fact that *AGO2* inactivation leads to these phenotypes in a background of wildtype *AGO1, AGO3,* and *AGO4*, which act redundantly in the miRNA pathway, suggests that slicing activity of AGO2 is important in development. Yet, biochemical or genetic evidence that slicing is required for the observed phenotypes is currently lacking. Evolutionary conservation of slicer activity would also be consistent with an antiviral function of AGO2 in mammals; this will be discussed in Section 5.

## 3. Innate Antiviral Immunity and the Interferon Pathway

**Figure 5:**
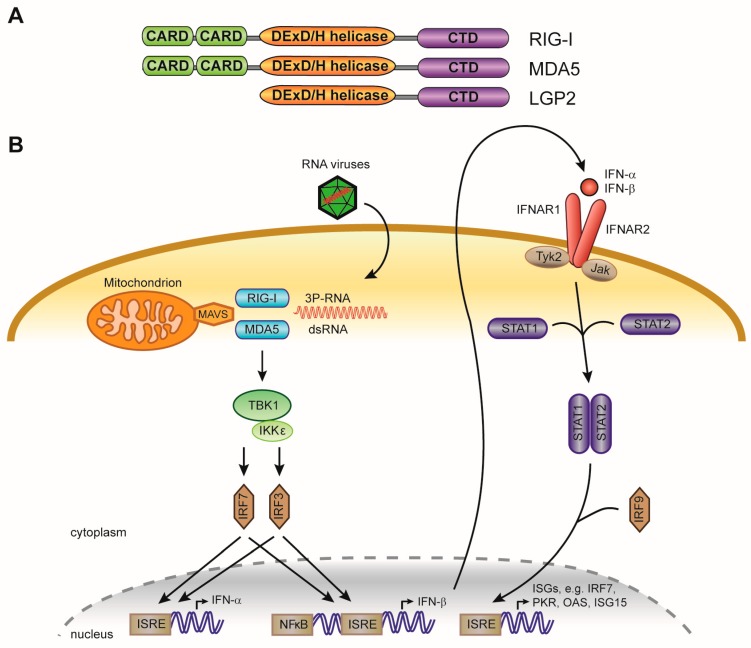
Cytosolic recognition of foreign nucleic acids and activation of interferon stimulated genes (ISGs). (**A**) Domain structure of the cytosolic RNA sensors RIG-I and MDA5, showing the CARD signaling domain, the DExD/H-box helicase domain, and the C terminal domain (CTD). LGP2 lacks the CARD signaling domain. (**B**) Schematic representation of the interferon response in mammals. RIG-I and MDA5 recognize non-self viral RNA signatures, including the presence of a 5’ triphosphate moiety on RNA (3P-RNA) or long dsRNA. Upon detection of foreign nucleic acids, the CARD domains transduce the signal to mitochondrial antiviral-signaling protein (MAVS) located at mitochondrial membranes, leading to the phosphorylation and activation interferon response factors (IRF) 3 and 7. Upon activation, IRF3 and IRF7 form homodimers and translocate to the nucleus, where they bind Interferon-Stimulated Response Elements (ISRE) to activate transcription of type I interferons (IFN-α and IFN-β). Type I IFNs translocate across the cell membrane, after which they signal in a paracrine or autocrine manner via the interferon-α/β receptor (consisting of two subunits, IFNAR1 and IFNAR2). This activates the JAK-STAT pathway, leading to phosphorylation of STAT transcription factors (signal transducer and activator of transcription). Phosphorylated STAT1 and STAT2 heterodimerize, and translocate to the nucleus to activate expression of broad range of ISGs.

The innate immune response to viral infection in mammals is characterized by induction of type I interferons (IFN), cytokines with strong antiviral activity [109]. They signal in an autocrine and paracrine fashion via the interferon-α/β receptor (IFNAR), consisting of two subunits, IFNAR1 and IFNAR2 [110,111,112] (Figure 5). Upon binding of type I IFNs, IFNAR activates the JAK-STAT pathway to induce expression of hundreds of Interferon stimulated genes (ISGs) [113,114]. Collectively, ISGs impede viral replication and provide a window for the adaptive immune response to clear the infection [115]. ISGs inhibit viral replication via a wide range of activities, including the inhibition of transcription (Mx1, TRIM5), translation (PKR, IFIT family members, OASL), and replication (IFIT family members, OAS1/2/3), and the induction of RNA degradation and apoptosis (RNaseL) [115,116]. Furthermore, type I IFNs can induce the release of chemokines, increase antigen presentation by innate immune cells, induce the production of antibodies, and stimulate effector T cell responses [117]. As the activities of ISGs are potentially cytotoxic, expression and activation of interferon and ISGs is under tight regulation. 

### 3.1. Sensing of Foreign Nucleic Acids

The innate immune system distinguishes self from non-self based on molecular patterns. Viral infection is sensed by the presence of foreign nucleic acids, either on the basis of non-self structural features, such as double-stranded RNA or the presence of a 5’ triphosphate moiety on RNA, or their subcellular localization (Figure 5A). Different receptors have been identified that recognize foreign DNA or RNA, including the RIG-I-like receptor (RLR) family of RNA sensors (retinoic acid inducible gene I [RIG-I; also known as DDX58]) and melanoma differentiation associated gene 5 [MDA5; also known as IFIH1]), members of the Toll-like receptor (TLR) family (specifically TLR3, TLR7, TLR8, and TLR9), and the DNA sensors absent in melanoma 2 (AIM2) and cyclic GMP-AMP synthetase (cGAS) [118]. Upon recognition of foreign nucleic acids, these pattern recognition receptors directly or indirectly activate transcription factors, including nuclear factor-κB (NF-κB), IFN-regulatory factor 3 (IRF3), and IFN-regulatory factor 7 (IRF7), subsequently leading to the induction of chemokines, cytokines, and antiviral effector proteins. 

Viral double-stranded RNA is also recognized by a group of ISGs that function as antiviral effectors, rather than as signaling molecules. Members of this group are dsRNA-activated protein kinase R (PKR; also known as eIF2AK2), 2’-5’-oligoadenylate synthetase 1 (OAS1), and adenosine deaminase acting on RNA 1 (ADAR1). For example, upon activation by dsRNA binding, PKR inhibits cap-dependent translation of (viral and host) mRNA [119]. Likewise, dsRNA activates OAS1, which synthesizes 2’-5’ oligomers of adenosine (2’-5’ oligoadenylate) [120,121]. The second messenger 2’-5’ oligoadenylate in turn activates ribonuclease L (RNase L) [122], which degrades viral and cellular RNAs. 

### 3.2. RIG-I-Like Receptors

The RLR protein family consists of the three members RIG-I, MDA-5, and LGP2, all of which are expressed in the cytosol of most cell types [123] (Figure 5A). Furthermore, RLRs are themselves ISGs that are transcriptionally induced by IFN in a positive-feedback loop [123]. RLR receptors are DExD/H-box helicases, where DExD/H refers to Asp-Glu-x-Asp/His and “x” can be any amino acid [124]. The conserved helicase core of RLRs, consisting of two highly similar tandem helicase domains (Hel1 and Hel2) separated by a unique insertion (Hel2i), is critical for the integration of signals triggered upon RNA binding [124]. The C-terminal domain (CTD) of RLRs is the site of RNA recognition and confers ligand specificity [125,126]. MDA5 and RIG-I, in contrast to LGP2, possess two adjacent CARD (caspase activation and recruitment) domains at their N-terminus. These domains are important for transmitting structural changes via the conserved helicase domains, leading to the oligomerization of the adaptor protein MAVS (mitochondrial antiviral signaling) on mitochondrial membranes [127]. This results in the activation of IRF-3, IRF-7 and NF-κB, which subsequently translocate to the nucleus and induce transcription of type I IFNs and a subset of ISGs [113,128,129]. 

While the lack of CARD domains renders LGP2 signaling incompetent, it is important for fine tuning the immune response. LGP2 amplifies signaling via MDA5 by promoting the rate of MDA5-RNA interactions and increasing nucleation of MDA5 filaments on dsRNA [130,131]. As a consequence, loss of LGP2, results in an increased sensitivity to viruses that are detected by MDA5, such as picornaviruses [132,133]. Likewise, signaling upon dsRNA detection by RIG-I is regulated by LGP2 [134,135]. In line with its role in modulating the immune response, mice deficient in LGP2 have altered CD8+ T cell responses to rabies virus, influenza A virus and West Nile virus infections [136,137,138]. 

In addition to their role in RNA sensing and signaling, RLRs may also exert direct antiviral activity [139,140]. For example, RIG-I binding to the 5’-ε region of the pre-genomic RNA of hepatitis B virus counteracts the interaction with the viral polymerase (P), resulting in suppression of viral replication [139]. Likewise, the influenza A virus nucleocapsid is destabilized by binding of RIG-I to the viral “panhandle” promoter, resulting in interference with virus replication at the onset of infection [140]. These findings suggest that RIG-I, besides its well-characterized role in innate sensing, possesses antiviral effector functions by directly interacting with viral dsRNA structures.

### 3.3. Innate Antiviral Immunity in Pluripotent Cells

Stem cells are critical for tissue maintenance, growth, and repair [141]. Stem cells reside in specific anatomical structures, called stem cell niches, in which they communicate with each other and their surrounding cells, and respond to cues received from the extracellular matrix [142]. Stem cell niches are often in close proximity to the blood supply [142], but tissue stem cells, such as smooth muscle progenitor cells and skeletal stem cells, may also circulate in the peripheral blood [143,144]. These characteristics render stem cells sensitive to infection by different pathogens. Given the importance of stem cells and their relatively low abundance, it is essential that effective antiviral defense mechanisms are in place to protect these cells from damage and cell death. Yet, a full-blown interferon response could be detrimental due to the potentially cytotoxic effector mechanisms induced. Indeed, the IFN response is severely attenuated in embryonic stem cells (ESCs) [145], and is regulated at multiple levels in human ESCs. For instance, pathogen recognition receptors including the dsRNA sensor and signaling molecules OAS1, MDA5 and TLR3, are downregulated in human ESCs [146], whereas other proteins such as PKR and RIG-I are expressed, but fail to respond to dsRNA stimulation [146]. Moreover, a recent study demonstrated that suppression of the IFN response in mESCs depends on miRNA mediated silencing of MAVS, and that knockout of a single miRNA (miR-673) restores the response [147]. 

Although stem cells are refractory to type I IFNs [145], human ESCs are highly resistant to viral infection [148]. It was found that human ESCs and induced pluripotent stem cells (iPSCs) intrinsically express a subset of ISGs, including IFITM1, IFITM3, EIF3L, and BST2, which conferred protection against infection with dengue virus and vesicular stomatitis virus [148]. Antiviral protection may extend to other virus families, as IFITM-family members restrict entry of diverse viruses, at the level of pH or cathepsin-dependent fusion in endo/lysosomes and to a lesser extent at the plasma membrane [149], although post-entry restriction mechanisms have been reported as well [149]. 

Intrinsic ISG expression varies between stem cell types including ESCs and tissue stem cells (mesenchymal stem cells, neural stem cells and pancreatic stem cells), and decreases upon stem cell differentiation [148,150]. The decreased basal ISG expression and increased responsiveness to IFN allow terminally differentiated cells to induce the full spectrum of antiviral ISGs. Interestingly, besides being IFN non-responsive and expressing high levels of selected ISGs, ESCs have been found to possess an active RNAi response, which may contribute to antiviral control [13] (discussed in Section 5.3).

### 3.4. Viral Antagonism of the IFN Response

The interferon system exerts strong adaptive pressure on viruses, which in turn have evolved antagonists of type I and type III IFN responses [151,152]. For example, the influenza A virus non-structural protein 1 (NS1) is a well-known IFN antagonist that inhibits the phosphorylation and nuclear import of IRF-3 and interferes with posttranscriptional processing of cellular antiviral pre-mRNAs via its dsRNA binding properties [153,154]. dsRNA-binding proteins from many other mammalian viruses, including vaccinia virus E3L, Ebola virus VP35, reovirus s3, bunyavirus NSs, and herpesvirus US11, likely bind viral dsRNA to prevent detection by cellular dsRNA sensors and induction of an IFN response [155]. Another mode of action is antagonism of IFN pathway components such as RLRs or downstream signaling components including IRF3, IRF7, STAT1, or STAT2 [152]. For example, the NS5 protein of yellow fever virus inhibits the antiviral action of the IFN pathway by binding to STAT2 [156], and thus preventing its interaction with the IFN-stimulated response element in the promoters of ISGs [156]. Many other mechanisms for IFN suppression have been described, including the regulation of phosphorylation events (e.g., Ebola virus VP40), interference with ubiquitin modification (e.g., influenza A virus NS1), host transcription shut-off (e.g., adenovirus E1A), inhibition of RNA processing and trafficking (e.g., vesicular stomatitis virus M protein), translational shut-off (e.g., hepatitis C virus), or IFN decoy mechanism (e.g., vaccinia virus B18R). For a review, see [152].

## 4. Antiviral RNA Interference in Insects

While small RNA pathways are evolutionarily conserved from invertebrates to mammals, animal clades differ in the specific makeup of these pathways. *Drosophila* provides a notable example, as the miRNA and siRNA pathways are largely independent pathways, with Dicer-1 and Argonaute1 (Ago1) dedicated to the miRNA pathway, and Dicer-2 and Argonaute2 (Ago2) dedicated to the siRNA pathway [157] (discussed in Section 2). This characteristic facilitates the use of genetic approaches to assess the antiviral function of RNAi, as *Dicer-2* and *Ago2* null mutants are viable and fertile, and do not have defects in the gene regulatory miRNA pathway. 

### 4.1. Broad Antiviral Function of Insect RNAi

Recognition of dsRNA as a danger signal to induce an immune response is an effective strategy, since almost all viruses produce dsRNA during their replication cycle [158,159]. Therefore, antiviral RNAi is broadly active against a large number of RNA and DNA viruses in insects. The most prominent viral dsRNA sources are (i) genomes of dsRNA viruses, (ii) replication intermediates of positive and negative sense RNA viruses, (iii) long fold-back structures in viral RNA, and (iv) convergent transcripts from the gene-dense genomes of DNA viruses (Figure 1) [11]. Dicer-2 recognizes and cleaves these viral dsRNA molecules into 21 nt viral vsiRNAs, which are loaded into Ago2-containing RISC complexes [55,160,161,162]. Whereas endogenous sources of siRNAs rely on both Dicer-2 co-factors R2D2 and Loqs-PD [35], vsiRNA biogenesis and RISC loading can occur in the absence of Loqs-PD [163]. Upon loading with vsiRNAs, RISC is programmed to specifically recognize and slice viral RNA, resulting in reduced viral replication [11,163,164,165,166]. The importance of RNAi in antiviral defense has been demonstrated using fly mutants deficient in *Dicer-2*, *R2D2*, and *Ago2*, which are more susceptible to virus infection and accumulate higher virus levels than wildtype flies [55,159,160,163,165,166,167]. Similarly, knockdown or inactivation of *Dicer-2* and *Ago2* in mosquitoes results in higher viral titers upon infection with different viruses [168,169,170,171]. Thus, antiviral RNAi targets viral RNA using two distinct steps, first by cleavage of dsRNA replication intermediates and second, through slicing of single-stranded RNAs by Ago2. 

Although the presence of two Dicer paralogs have facilitated genetic dissection of antiviral RNAi in flies, multiple Dicers are not required *per se* for a functional antiviral RNAi response. For example, the nematode *C*. *elegans* encodes only a single Dicer that produces both miRNAs and siRNAs, yet RNAi has antiviral activity against non-natural and natural viruses [172,173,174]. Another point emerging from a cross-species comparison relates to the role of RNA-dependent RNA polymerases (RdRPs) in antiviral RNAi. Cellular RdRPs contribute to the amplification of RNAi and the establishment of a systemic response in plants and nematodes through dsRNA synthesis used for the production of secondary siRNAs [175]. Insects do not encode RdRP genes, yet it has been proposed that a systemic immune response is established through spread of the RNAi signal to non-infected cells [176]. This occurs via a non-conventional mechanism in which viral RNA is reverse transcribed into viral DNA by cellular retrotransposons, followed by transcription of these viral DNA forms for de novo synthesis of secondary siRNAs [177,178,179].

### 4.2. Viral Suppressors of RNAi

Insect viruses are not defenseless against the activity of the RNAi pathway; many have developed strategies to antagonize the production or activity of vsiRNAs by expressing viral suppressors of RNAi (VSR) [11,180,181]. Numerous VSRs have been identified and the mechanisms by which they suppress RNAi is diverse, for instance by sequestering dsRNA or vsiRNAs to prevent Dicer-2 processing or by inhibiting Ago2 function through direct interaction with the RISC complex. The fact that viruses, which normally strive to reduce genome size to a minimum, devote genomic space to VSRs underscores the strength of RNAi as an antiviral mechanism in insects.

One of the best characterized VSRs is the Flock House virus (FHV) B2 protein, which is able to inhibit RNA silencing both in animals and plants [182], indicative of interference at evolutionarily conserved features [182]. The B2 protein binds long and short dsRNA in a sequence independent manner [174,183,184], thereby acting both upstream and downstream of Dicer cleavage. In addition, it has been shown that FHV B2 binds to Dicer-2, which could impede its processing activity [185]. The importance of this suppressor for viral replication in vivo has been demonstrated in several studies [55,164,186]. FHV mutants deficient in B2 expression (ΔB2) were found to have severe replication defects in RNAi-competent *Drosophila*, but not in RNAi-deficient flies, providing strong genetic support that RNAi-suppression is the main function of B2 [55,164,186]. 

VSRs have also been identified in members of other insect virus families. These include the Drosophila C virus 1A protein, a protein that binds dsRNA and thereby inhibits Dicer-2 processing [166], and Drosophila X virus and Culex Y virus VP3, both with dsRNA and siRNA binding activities [187]. Nora virus VP1 and cricket paralysis virus (CrPV) 1A antagonize the catalytic activity of Ago2, highlighting the importance of target RNA slicing for antiviral defense [55,188,189,190]. Suppression of RNAi has also been demonstrated for viruses that are transmitted by insects. For example, the capsid protein of yellow fever virus suppresses RNAi by inhibiting the processing of long dsRNA by Dicer-2 in the mosquito *Aedes aegypti* [171]. 

An evolutionary arms race may exist between host antiviral immune pathways and viral counter-defense mechanisms. Indeed, the existence of VSRs in insect viruses is indicative of the strong evolutionary pressure exerted by the RNAi pathway. VSRs, in turn, may drive adaptations in host genes, which could explain the observation that RNAi genes are among the fastest evolving genes in the *Drosophila* genome [191]. An ongoing cycle of adaptation and counter-adaptation could lead to host specificity of VSRs, as has been observed for the VP1 protein of Drosophila immigrans Nora-like virus [190].

## 5. Antiviral RNAi in Mammals

In contrast to *Drosophila*, in which the miRNA and siRNA pathways rely on distinct Dicer and Argonaute proteins [157], mammals only encode a single Dicer protein, which processes both miRNAs and siRNAs [192]. Moreover, a single AGO protein (AGO2) is capable of slicing target RNA, but it also mediates miRNA-dependent gene silencing [85,86,193]. This complicates the interpretation of experiments analyzing viral virus replication in conditions in which *Dicer* or *AGO* genes are inactivated. Yet, several genetic tools have been generated to study mammalian RNAi. For example, it has been possible to engineer mESCs lacking Dicer, which supported its essential role in miRNA biogenesis [194,195,196]. As differentiation of ESCs is dependent on cellular miRNAs [197,198], the generation of Dicer-deficient mouse embryonic fibroblasts required the use of conditional Cre-recombinase-mediated knockout [196,199,200]. In addition, an easy-to-manipulate human 293T cell line lacking human Dicer (NoDice cells) was generated using transcription activator-like endonucleases (TALENs). As expected, these cells were unable to process pre-miRNA precursors and lacked endogenous miRNA expression [201]. As in insects, additional to genetics approaches, the detection of vsiRNAs and identification of VSRs in mammalian viruses are used to study the antiviral function of RNAi.

### 5.1. Viral Small RNA Profiles

A direct approach to analyze RNAi-mediated targeting of viruses is by the detection of vsiRNAs using next-generation deep-sequencing technologies. Typically, small RNAs in the size range of 18–25/30 nt are purified and subjected to deep sequencing, after which the obtained small RNA sequences are analyzed using bioinformatics tools. Mammalian Dicer produces both ~22 (+/- 1 nt) miRNAs and siRNAs. Hence, the detection of vsiRNAs of ~22 nt in length, derived from the positive and negative strands at approximately equal ratios, as seen in invertebrates [10,11], would be indicative of viral dsRNA targeting by Dicer. 

Several studies failed to identify vsiRNAs in virus infected mammalian cells [16,17,18,19,202] (Table 1). For example, no detectable levels of vsiRNAs were obtained from the human Huh7 cell line infected with dengue virus (DENV) and West Nile virus (WNV) [203]. Viral small RNAs displayed a broad size range between 17–29 nt in DENV infected cells and the predominant read length in WNV-infected cells were <20 nt, arguing against Dicer-mediated processing of viral dsRNA [203]. In agreement, a broad panel of viruses (DENV, WNV, yellow fever virus, Sindbis virus, Venezuelan equine encephalitis virus, measles virus, influenza A virus, reovirus, vesicular stomatitis virus, HIV-1, or herpes simplex virus-1) did not replicate at higher levels in NoDice cells than in Dicer competent cells [203]. Other studies identified detectable levels of viral small RNAs, although deletion of Dicer did not affect the levels of these small RNA and it has been suggested that they are generated by ISGs, such as RNaseL [204,205]. In contrast to these results, several recent studies have proposed that antiviral RNAi is functional in mammalian cells under specific cellular or experimental conditions. Specifically, antiviral RNAi was observed in differentiated cells using viruses that are VSR defective, in pluripotent stem cells, or under conditions in which the interferon response in inactivated. These studies are discussed in the following sections. 

### 5.2. Viral Suppressors of RNAi in Mammalian Viruses

The presence of virus-encoded suppressors in insect and plant viruses provides strong support for the antiviral potential of RNAi [11]. RNAi reporter assays and biochemical assays are often used for their identification and elucidation of the mechanisms of action. Strongest support for their importance in RNAi suppression in vivo, however, depends on the observation of severe replication defects of virus mutants lacking a putative VSR and rescue of this defect in RNAi-defective cells, as has been extensively analyzed for FHV ΔB2 in insects [55,164,186] (described in Section 4.2).

RNAi suppressive activity has now been reported for a number of proteins from mammalian viruses, including the B2 protein of Nodamura virus, NS1 of influenza A virus, the 3A protein of human enterovirus 71 (HEV71), Ebolavirus VP35, SARS coronavirus N protein, and yellow fever virus capsid [14,15,171,206,211,212] (Table 2). Several of these VSRs, including Nodamura virus B2, influenza A virus NS1, and Ebolavirus VP35, are dsRNA binding proteins that possess a dual function in suppressing both the IFN pathway and RNAi [211,213,214,215,216]. The yellow fever virus capsid protein has likewise been proposed to bind dsRNA and prevent Dicer processing, although this has thus far only been studied in the mosquito *Aedes aegypti* [171]. In addition, the subgenomic flavivirus RNA (sfRNA), which is produced by incomplete digestion of the 3’UTR by the exonuclease Xrn-1 [217], has been proposed as an suppressor RNAi [218], although many other activities has been attributed to sfRNA, including the modulation of host antiviral responses by antagonizing G3BP1, G3BP2, and CAPRIN1 [219]. A unique mechanism for RNAi suppression was found for the poly(A) polymerase of vaccinia virus (VP55), which inhibits the stability of small RNAs, in particular miRNAs [220]. VP55 mediates the addition of 2-7 adenosines at the 3’ end of AGO-bound miRNAs (tailing), resulting in their degradation [220]. 

For many of the proposed mammalian VSRs, the physiological importance during virus infection awaits elucidation. A notable exception is the B2 protein of Nodamura virus, like FHV a member of the family *Nodaviridae*. In a somatic cell line (BHK-21) and the limbs of newborn mice, vsiRNAs were detected upon infection with a Nodamura virus mutant lacking B2 (ΔB2), but not upon infection with wildtype virus [14]. Moreover, abolishing the RNAi suppressive activity of B2 rendered suckling mice resistant to infection, whereas wild-type Nodamura virus caused a lethal infection [14]. In agreement, Nodamura virus ΔB2 mutants showed a broad range of replication defects in different mammalian cell lines, suggestive of cell specific differences in RNAi potency [221]. Together, these findings illustrate that suppression by VSRs can mask the antiviral activity of RNAi and that inactivation of VSRs may be required to detect antiviral RNAi in differentiated mammalian cells. 

Another VSR that has been proposed to veil antiviral RNAi is influenza A virus NS1. It has been demonstrated that an influenza A virus mutant lacking NS1 (ΔNS1) replicates only poorly, indicating that NS1 is essential for successful infection [229]. The same study found that this replication defect was rescued in cells deficient in PKR, an important effector of the IFN pathway [229], suggesting that evasion of the IFN response is the major function of NS1 in this experimental system. Could there still be a role for NS1 in suppression of RNAi? Intriguingly, the slicing activity of AGO2 was reported to restrict influenza A virus replication in somatic cells, independent of interferon [15], although another study found that influenza virus (both wildtype virus and ΔNS1 mutant), accumulated to similar levels in mouse embryonic fibroblasts (MEFs) with or without an intact RNAi pathway [21]. Small RNA deep sequencing identified vsiRNAs in influenza A virus ΔNS1 infection, but not in wildtype influenza A virus infections, supporting a physiological role of NS1 in masking the antiviral RNAi pathway [15]. Importantly, vsiRNA production was lost in Dicer-deficient 293T cells, but rescued upon ectopic expression of human Dicer, providing genetic support that the detected vsiRNAs were Dicer dependent [15]. These observations have been confirmed in a study using wildtype and ΔNS1 mutant influenza A virus in 293T cells and the lung epithelial cell line A549 [207]. However, these vsiRNAs did not affect viral gene expression in this study, likely caused, at least partially, by inefficient vsiRNA loading into RISC [207]. Yet, earlier studies reporting that NS1 does not inhibit RNAi in mammals using reporter assays or engineered viruses [230,231,232,233,234,235]. The discrepancy between studies that did not detect vsiRNAs [202,208] and recent reports that did detect vsiRNAs in influenza A virus infection may be partially explained by the use of influenza ΔNS1 mutants [15,207] and AGO2 immunoprecipitation before deep sequencing [15]. The sensitivity to detect rare vsiRNAs may be increased by enrichment for siRNAs bound to AGO2, rather than sequencing all small RNAs, which would also detect random degradation products. 

The notion that VSRs may mask antiviral RNAi in mammals has now been supported by a third example, human enterovirus 71 (HEV71), an RNA virus from the family *Picornaviridae*. Specifically, the 3A protein of HEV71 was found to suppress the RNAi response by binding long dsRNA as determined by gel mobility shift assay and Northern blot [206]. Viral siRNAs were detected in 293T cells and mice upon infection with virus mutants carrying two mutations (D23A or R34A) in 3A, but not in infections with wildtype virus [206]. In agreement, mutant virus had severe replication defects, which was rescued in Dicer-deficient cells, albeit slightly [206]. 

### 5.3. Antiviral RNAi in Pluripotent Cells

Major differences exist in the innate immune response between pluripotent and differentiated cells [148] (discussed in Section 3.3), and RNAi has recently been proposed as an additional antiviral mechanism in mESCs [13]. Specifically, vsiRNAs with the characteristics of canonical siRNAs were detected upon infection with encephalomyocarditis virus (EMCV), the level of which substantially decreased upon cell differentiation [13]. Likewise, vsiRNAs were observed upon infection of mESCs with the Nodamura virus ΔB2 mutant, whereas wildtype virus failed to produce vsiRNAs [13,14]. Extending these observations to the human system, it was found that Zika virus infection of human neural progenitor cells results in vsiRNA production, and accordingly that *Dicer* and *AGO2* knockdown or transgenic expression of Nodamura virus B2 increases viral RNA levels [210]. Together, these studies suggest that Dicer-mediated processing of dsRNA replication intermediates into vsiRNAs may occur in pluripotent stem cells, but not or less efficiently in differentiated cells. 

Several non-mutually exclusive possibilities have been proposed to explain why RNAi might be favored over the IFN response in stem cells [236]. Pluripotent cells readily produce triggers of the IFN response (e.g., cytoplasmic dsRNA) [237], but the IFN response may be incompatible with pluripotency. For example, pluripotent cells undergo rapid cell division and the IFN response might be muted to prevent the associated antiproliferative effects [238]. In addition, as interferon stimulates differentiation, controlled inhibition of the IFN response in pluripotent cells might serve as a means to maintain potency [238]. An effective RNAi response, on the other hand, seems to be important for stem cell biology as maintenance of stem cell properties is regulated by the endogenous RNAi machinery [239]. Furthermore, high levels of transposon activity have been reported in undifferentiated cells [240] and RNAi likely contributes to transposon repression in these cells. 

Dicer is crucial for processing long dsRNA into siRNAs, raising the possibility that RNAi activity is regulated at the level of Dicer in different cell types. For example, the activity of human Dicer is dependent on the substrate, as demonstrated by different cleavage rates on pre-miRNAs and long dsRNA [241,242], suggesting different modes of recognition and/or processing of miRNA and siRNAs in vivo. Furthermore, the N-terminal helicase domain of mammalian Dicer was found to autoinhibit siRNA processing activity [241,243]. In agreement, in human somatic cells ectopically expressing an N-terminally truncated version of human Dicer, vsiRNAs were detected upon influenza A virus infection [208]. This is analogous to a natural situation in mice oocytes, which naturally express an N-terminally truncated isoform of Dicer, Dicer-O, which efficiently processes long dsRNA substrates into siRNAs [243]. Expression of Dicer-O is driven by an insertion in intron 6 of an MT-C retroelement [243], a long terminal repeat (LTR) retrotransposon that is active in mouse oocytes and can be used as alternative promoters for adjacent genes [244]. The MT-C promoter drives expression of an alternative exon (AltE) [244] in-frame with the next exon, resulting in the deletion of the N-terminal DExD helicase domain in the Dicer-O variant [243]. The truncated Dicer-O isoform is limited to the *Muridae* family of rodents [243], and it is unlikely to play a role in antiviral RNAi. Yet, these observations [208,243] suggest an important role for Dicer processivity in antiviral RNAi. Indeed, it was recently demonstrated that the antiviral Dicer of *Drosophila*, Dicer-2, has two distinct modes to process dsRNA substrates. Substrates with 3’ overhangs are cleaved distributively, whereas blunt dsRNA is locally unwound, threaded through the helicase domain, and processively cleaved in an ATP dependent manner [40] (Figure 2C). Distributive cleavage of substrates with 3’overhangs seems consistent with the main function of human Dicer in miRNA biogenesis. Moreover, although the helicase domain is conserved in human Dicer, a dsRNA threading mechanism has thus far not been reported. Thus, whether the threading mechanism of Dicer is essential for efficient antiviral RNAi, and whether human Dicer adopts this mechanism under specific cellular conditions remains an interesting question for future research [40].

### 5.4. RNA Interference and Interferon Pathway

In mammals, the potent IFN response is the main innate antiviral pathway [113,118], whereas RNAi seems to be antiviral in undifferentiated cells in which the IFN system is not active [13,146]. This raises the question whether the IFN response masks or inhibits antiviral RNAi in mammals. In agreement with this hypothesis is the observation that in MEFs deficient in the signaling molecule MAVS or interferon receptor (IFNAR1), defective in respectively sensing non-self RNA and responding to type I IFNs, long dsRNA induces sequence-specific gene silencing in a Dicer and AGO2-dependent manner [21]. Although the authors did not directly assess vsiRNA production using a deep-sequencing approach in these cells, antiviral RNAi activity was suggested via a “dsRNA vaccination experiment” in which IFN-deficient cells were protected from virus infection by prior treatment with virus sequence-specific long dsRNA [21]. Although these experiments suggest competition between the IFN response and RNAi, HeLa cells deficient in both RIG-I and MDA5 did not produce detectable levels of vsiRNAs upon infection with Sindbis virus, yellow fever virus, and Coxsackie virus B3 [17]. 

Support for an interaction between the RNAi and IFN pathways also emerged from a large-scale proteomics study to identify interactors of the innate immune system. This study identified a physical interaction of LGP2 and Dicer [245], which was subsequently shown to contribute to suppression of the RNAi pathway [20]. Binding of LGP2 to Dicer prevented cleavage of long dsRNA into siRNAs in vitro and ectopic expression of LGP2 in *IFNAR1^-/-^* cells inhibited RNAi, as shown by northern blots detecting siRNAs derived from transfected dsRNA [20]. Knock-out of *LGP2* resulted in stronger RNAi responses in reporter assays, but the effect was less pronounced than in cells lacking *IFNAR1* [20]. Moreover, it was found that LGP2 binds dsRNA binding sites on the Dicer co-factor TRBP, thereby inhibiting pre-miRNA binding and maturation [246]. Together, these results suggest that LGP2 interferes with RNA silencing, strengthening the notion that the IFN response masks or inhibits an antiviral RNAi response in differentiated mammalian cells. 

Reciprocally, RNA silencing may also play a regulatory role in the interferon response. Several studies have proposed that miRNAs play important functions in the negative regulation of cytotoxic ISGs, particularly those associated with cell proliferation and cell death [247,248,249,250,251]. Moreover, viral infection or treatment with the dsRNA mimic poly I:C induced ADP-ribosylation of AGO2, resulting in inhibition of RISC activity and, thus, decreased siRNA and miRNA silencing activity [247]. As many ISGs are targets of miRNAs, inhibition of RISC may increase ISG expression, suggesting that this mechanism contributes to the rapid ISG expression upon viral infection [247]. 

## 6. Summary and Open Questions

Vertebrates rely on the protein-based IFN response to combat viral infections, whereas the RNAi machinery, known for its potent antiviral activity in invertebrates, is conserved but primarily functions in gene regulation. Mammals encode a single Dicer protein and four AGO proteins, of which only AGO2 is slicer competent. The function of these proteins in both the miRNA and siRNA pathways makes it difficult to genetically dissect the role of the RNAi pathway in inhibiting viral replication. The notion that both the IFN response and RNAi rely on dsRNA to initiate the antiviral response adds another level of complexity. 

With the advancement of next generation sequencing technologies, a growing body of evidence has emerged that supports a role for RNAi in antiviral defense in mammals. Detection of canonical vsiRNAs in ESCs infected with EMCV or Nodamura virus ΔB2 provided the first compelling evidence for a role of antiviral RNAi in mammals [13,14]. ESCs possess an attenuated immune response [145], caused by reduced gene expression of IFN-pathway components or, in some instances, failure to respond to dsRNA triggers [146]. It is now apparent that RNAi is suppressed by the IFN pathway, likely due to the action of one or more ISGs [21] and through the interaction of Dicer and LGP2 [20]. 

VSRs seem to play an important role in differentiated cells, demonstrated by the accumulation of vsiRNAs during Nodamura virus ΔB2 and HEV71 3A mutant virus infections [13,14,206]. These findings were complemented by the detection of AGO2-associated siRNAs in somatic cells infected with Influenza A virus ΔNS1 [15]. These studies indicate that VSRs may mask the antiviral RNAi response in mammals. This situation is markedly different from the situation in plants and insects, in which vsiRNAs are readily detected with most, if not all wildtype viruses analyzed, hinting at differences in processivity of Dicer enzymes or differences in accessibility of viral dsRNA in mammals and insects. 

**Figure 6:**
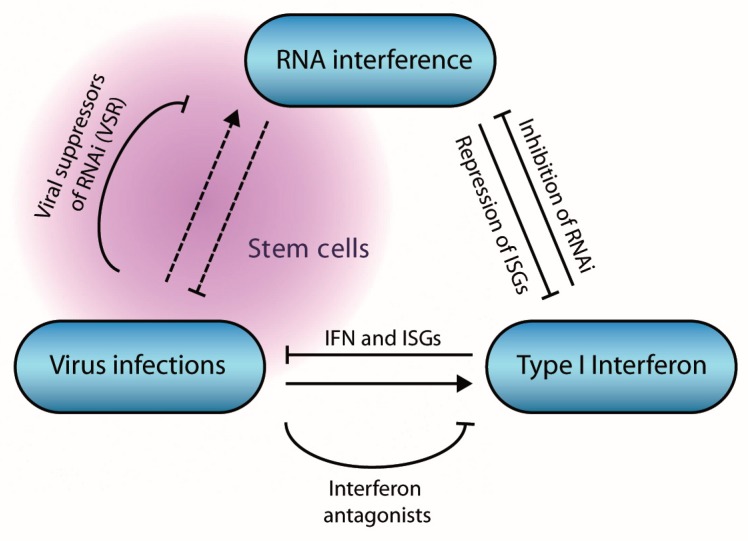
Interactions between viruses, RNA interference (RNAi), and the interferon pathway in mammals. Virus infection induces the expression of type I interferons, leading to the expression of Interferon stimulated genes (ISGs) that collectively restrict virus infection. The interferon pathway inhibits RNAi via multiple mechanisms, whereas miRNAs inhibit expression of ISGs. Virus infection induces an antiviral RNAi response under specific conditions, in stem cells or in absence of viral suppressors of RNAi.

Antiviral RNAi thus seems to be affected by the cellular context, IFN responses, and viral counter-defense mechanisms (Figure 6). Important questions still remain for each of these aspects. (i) How does the cellular context affect the antiviral immune response? Are there tissue and cell type specific differences in antiviral RNAi? Why is the antiviral RNAi pathway functional in stem cells and why is this activity lost upon differentiation? For example, are there specific determinants in stem cells that favor RNAi over the IFN response? How does cell potency (e.g., toti-, pluri-, and multipotency) affect the dominant antiviral immune response, and do tissue stem cells use RNAi for antiviral defense? (ii) Which factors, beyond LGP2, contribute to the inhibition of RNAi in differentiated cells? What are the relative contributions of the IFN and RNAi responses to host defense. (iii) How widespread is RNAi suppression among mammalian viruses? Do mammalian viruses encode VSRs that suppress AGO2, and what is the course of infection of virus mutants lacking this activity? Answers to these questions will shed light on the sophisticated RNAi pathway and its functions in antiviral defense. 

## Figures and Tables

**Table 1:** Recent studies reporting viral small RNA profiles in mammalian experimental systems.

Virus (Mutant)	Virus Family	Host	Experimental System	Approach	Viral siRNAs Detected ^a^	Reference
Nodamura virus (NoV ∆B2)	Nodaviridae	mice	Suckling mice	total small RNA	yes	[14]
		hamster	BHK cells	total small RNA	yes	[14]
		mice	Embryonic stem cells	total small RNA	yes	[13]
Encephalomyocarditis virus	Picornaviridae	mice	Embryonic stem cells	total small RNA	yes	[13]
Human enterovirus 71 (HEV71 D23A)	Picornaviridae	human	293T cells	total small RNA	yes	[206]
Influenza A virus (∆NS1)	Orthomyxoviridae	human	A549 cells	total small and AGO2 associated small RNAs	yes	[15]
		human	A549 cells	total small RNA	yes	[207]
		human	293T cells	total small RNA	yes	[207]
Influenza A virus		human	293T cells	total small RNA	no	[208]
		mouse	MEFs	total small RNA	no	[202]
		human	A549 cells	total small RNA	no	[207]
		human	293T cells	total small RNA	no	[207]
Borna disease virus	Bornaviridae	rat	C6 glioma cells	total small RNA	no	[202]
Coxsackie virus B3	Picornaviridae	human	HeLa cells	total small RNA	no	[17]
Sindbis virus	Togaviridae	human	HeLa cells	total small RNA	no	[17]
		mice	MEFs	total small RNA	no	[202]
		human	HEK293 cells	total small RNA	no	[209]
		human	HEK293 cells	total small RNA	no	[205]
		monkey	Vero cells	total small RNA	no	[205]
Vesicular stomatitis virus	Rhabdoviridae	mice	MEFs	total small RNA	no	[202]
Yellow fever virus	Flaviviridae	human	HeLa cells	total small RNA	no	[17]
Zika virus	Flaviviridae	human	Neural progenitor cells	total small RNA	yes	[210]

MEFs, mouse embryonic fibroblasts; ^a^ Defined as a peak of 22-nt (+/- 1 nt) in size profiles of viral small RNAs, derived from both positive and negative viral RNA strands.

**Table 2:** Examples of viral suppressors of RNAi in mammalian (arbo-)viruses.

Virus	VSR	Mechanism RNAi Suppression	IFN Antagonist	Mechanism IFN Antagonism	References
Ebola virus	VP35	dsRNA binding	Yes	Inhibits PACT-induced RIG-I ATPase activity; Blocks phosphorylation of IRF-3 and production of IFN-β	[222,223]
Human enterovirus 71	3A	dsRNA binding	Yes (minor)	nt	[206]
SARS coronavirus	N	dsRNA binding	Yes	Inhibits TRIM25-mediated RIG-I ubiquitination, resulting in inhibition of IFN production	[212,224]
Influenza A virus	NS1	dsRNA binding	yes	Inhibits the ubiquitin ligase TRIM25, responsible for ubiquitination and activation of RIG-I; Inhibits PKR; Prevents activation of the 2′-5′ oligoadenylate synthetase/RNase L system	[225,226,227]
Nodamura virus	B2	dsRNA binding	nt	-	[228]
Vaccinia virus	VP55	Polyadenylation of miRNAs, leading to their degradation	no	-	[220]
Yellow fever virus	capsid	dsRNA binding ^a^	no	-	[171]

nt, not tested. ^a^ RNAi suppression was shown in mosquitoes, but not analyzed in mammals.

## References

[1] Fire A., Xu S., Montgomery M.K., Kostas S.A., Driver S.E., Mello C.C. (1998). Potent and specific genetic interference by double-stranded RNA in Caenorhabditis elegans. Nature.

[2] Ghildiyal M., Zamore P.D. (2009). Small silencing RNAs: An expanding universe. Nat. Rev. Genet..

[3] Schirle N.T., Kinberger G.A., Murray H.F., Lima W.F., Prakash T.P., MacRae I.J. (2016). Structural analysis of human Argonaute-2 bound to a modified siRNA guide. J. Am. Chem. Soc..

[4] Elkayam E., Kuhn C.D., Tocilj A., Haase A.D., Greene E.M., Hannon G.J., Joshua-Tor L. (2012). The structure of human argonaute-2 in complex with miR-20a. Cell.

[5] Ketting R.F. (2011). The many faces of RNAi. Dev. Cell.

[6] Carthew R.W., Sontheimer E.J. (2009). Origins and mechanisms of miRNAs and siRNAs. Cell.

[7] Baulcombe D. (2004). RNA silencing in plants. Nature.

[8] Hamilton A.J., Baulcombe D.C. (1999). A species of small antisense RNA in posttranscriptional gene silencing in plants. Science.

[9] Ratcliff F., Harrison B.D., Baulcombe D.C. (1997). A similarity between viral defense and gene silencing in plants. Science.

[10] Ding S.W., Voinnet O. (2007). Antiviral immunity directed by small RNAs. Cell.

[11] Bronkhorst A.W., van Rij R.P. (2014). The long and short of antiviral defense: Small RNA-based immunity in insects. Curr. Opin. Virol..

[12] Dang Y., Yang Q., Xue Z., Liu Y. (2011). RNA interference in fungi: Pathways, functions, and applications. Eukaryot. Cell.

[13] Maillard P.V., Ciaudo C., Marchais A., Li Y., Jay F., Ding S.W., Voinnet O. (2013). Antiviral RNA interference in mammalian cells. Science.

[14] Li Y., Lu J., Han Y., Fan X., Ding S.W. (2013). RNA interference functions as an antiviral immunity mechanism in mammals. Science.

[15] Li Y., Basavappa M., Lu J., Dong S., Cronkite D.A., Prior J.T., Reinecker H.C., Hertzog P., Han Y., Li W.X. (2016). Induction and suppression of antiviral RNA interference by influenza A virus in mammalian cells. Nat. Microbiol..

[16] Lin Y.T., Kincaid R.P., Arasappan D., Dowd S.E., Hunicke-Smith S.P., Sullivan C.S. (2010). Small RNA profiling reveals antisense transcription throughout the KSHV genome and novel small RNAs. RNA.

[17] Schuster S., Tholen L.E., Overheul G.J., van Kuppeveld F.J.M., van Rij R.P. (2017). Deletion of cytoplasmic double-stranded RNA sensors does not uncover viral small interfering rna production in human cells. mSphere.

[18] Pfeffer S., Sewer A., Lagos-Quintana M., Sheridan R., Sander C., Grasser F.A., van Dyk L.F., Ho C.K., Shuman S., Chien M. (2005). Identification of microRNAs of the herpesvirus family. Nat. Methods.

[19] Umbach J.L., Cullen B.R. (2009). The role of RNAi and microRNAs in animal virus replication and antiviral immunity. Genes Dev..

[20] Van der Veen A.G., Maillard P.V., Schmidt J.M., Lee S.A., Deddouche-Grass S., Borg A., Kjaer S., Snijders A.P., Reis e Sousa C. (2018). The RIG-I-like receptor LGP2 inhibits Dicer-dependent processing of long double-stranded RNA and blocks RNA interference in mammalian cells. EMBO J..

[21] Maillard P.V., Van der Veen A.G., Deddouche-Grass S., Rogers N.C., Merits A., Reis e Sousa C. (2016). Inactivation of the type I interferon pathway reveals long double-stranded RNA-mediated RNA interference in mammalian cells. EMBO J..

[22] Cerutti H., Casas-Mollano J.A. (2006). On the origin and functions of RNA-mediated silencing: From protists to man. Curr. Genet..

[23] Miesen P., Joosten J., van Rij R.P. (2016). PIWIs go viral: Arbovirus-derived piRNAs in vector mosquitoes. PLoS Pathog..

[24] Bernstein E., Caudy A.A., Hammond S.M., Hannon G.J. (2001). Role for a bidentate ribonuclease in the initiation step of RNA interference. Nature.

[25] Elbashir S.M., Lendeckel W., Tuschl T. (2001). RNA interference is mediated by 21- and 22-nucleotide RNAs. Genes Dev..

[26] Elbashir S.M., Martinez J., Patkaniowska A., Lendeckel W., Tuschl T. (2001). Functional anatomy of siRNAs for mediating efficient rnai in Drosophila melanogaster embryo lysate. EMBO J..

[27] Zhang H., Kolb F.A., Jaskiewicz L., Westhof E., Filipowicz W. (2004). Single processing center models for human Dicer and bacterial RNase III. Cell.

[28] Miyoshi K., Tsukumo H., Nagami T., Siomi H., Siomi M.C. (2005). Slicer function of Drosophila argonautes and its involvement in RISC formation. Genes Dev..

[29] Matranga C., Tomari Y., Shin C., Bartel D.P., Zamore P.D. (2005). Passenger-strand cleavage facilitates assembly of siRNA into Ago2-containing RNAi enzyme complexes. Cell.

[30] Rand T.A., Petersen S., Du F., Wang X. (2005). Argonaute2 cleaves the anti-guide strand of siRNA during RISC activation. Cell.

[31] Liu Y., Ye X., Jiang F., Liang C., Chen D., Peng J., Kinch L.N., Grishin N.V., Liu Q. (2009). C3PO, an endoribonuclease that promotes RNAi by facilitating RISC activation. Science.

[32] Liu Q., Rand T.A., Kalidas S., Du F., Kim H.E., Smith D.P., Wang X. (2003). R2D2, a bridge between the initiation and effector steps of the Drosophila RNAi pathway. Science.

[33] Liu X., Jiang F., Kalidas S., Smith D., Liu Q. (2006). Dicer-2; R2D2 coordinately bind siRNA to promote assembly of the siRISC complexes. RNA.

[34] Tomari Y., Matranga C., Haley B., Martinez N., Zamore P.D. (2004). A protein sensor for siRNA asymmetry. Science.

[35] Marques J.T., Kim K., Wu P.H., Alleyne T.M., Jafari N., Carthew R.W. (2010). Loqs and R2D2 act sequentially in the siRNA pathway in Drosophila. Nat. Struct. Mol. Biol..

[36] Sabin L.R., Zhou R., Gruber J.J., Lukinova N., Bambina S., Berman A., Lau C.K., Thompson C.B., Cherry S. (2009). Ars2 regulates both miRNA- and siRNA- dependent silencing and suppresses RNA virus infection in Drosophila. Cell.

[37] Iwasaki S., Kobayashi M., Yoda M., Sakaguchi Y., Katsuma S., Suzuki T., Tomari Y. (2010). Hsc70/hsp90 chaperone machinery mediates ATP-dependent RISC loading of small RNA duplexes. Mol. Cell.

[38] Miyoshi T., Takeuchi A., Siomi H., Siomi M.C. (2010). A direct role for HSP90 in pre-RISC formation in Drosophila. Nat. Struct. Mol. Biol..

[39] Horwich M.D., Li C., Matranga C., Vagin V., Farley G., Wang P., Zamore P.D. (2007). The Drosophila RNA methyltransferase, DmHen1, modifies germline piRNAs and single-stranded siRNAs in RISC. Curr. Biol..

[40] Sinha N.K., Iwasa J., Shen P.S., Bass B.L. (2018). Dicer uses distinct modules for recognizing dsRNA termini. Science.

[41] Liu Z., Wang J., Cheng H., Ke X., Sun L., Zhang Q.C., Wang H.W. (2018). Cryo-EM structure of human dicer and its complexes with a pre-miRNA substrate. Cell.

[42] Krieger E., Vriend G. (2014). Yasara view-molecular graphics for all devices-from smartphones to workstations. Bioinformatics.

[43] Ma J.B., Yuan Y.R., Meister G., Pei Y., Tuschl T., Patel D.J. (2005). Structural basis for 5’-end-specific recognition of guide RNA by the A. fulgidus PIWI protein. Nature.

[44] Rand T.A., Ginalski K., Grishin N.V., Wang X. (2004). Biochemical identification of Argonaute 2 as the sole protein required for RNA-induced silencing complex activity. Proc. Natl. Acad. Sci. USA.

[45] Nakanishi K., Weinberg D.E., Bartel D.P., Patel D.J. (2012). Structure of yeast argonaute with guide RNA. Nature.

[46] Nowotny M., Gaidamakov S.A., Crouch R.J., Yang W. (2005). Crystal structures of RNase H bound to an RNA/DNA hybrid: Substrate specificity and metal-dependent catalysis. Cell.

[47] Faehnle C.R., Elkayam E., Haase A.D., Hannon G.J., Joshua-Tor L. (2013). The making of a slicer: Activation of human Argonaute-1. Cell Rep..

[48] Orban T.I., Izaurralde E. (2005). Decay of mRNAs targeted by RISC requires XRN1, the ski complex, and the exosome. RNA.

[49] Schirle N.T., MacRae I.J. (2012). The crystal structure of human argonaute2. Science.

[50] Czech B., Malone C.D., Zhou R., Stark A., Schlingeheyde C., Dus M., Perrimon N., Kellis M., Wohlschlegel J.A., Sachidanandam R. (2008). An endogenous small interfering RNA pathway in Drosophila. Nature.

[51] Ghildiyal M., Seitz H., Horwich M.D., Li C., Du T., Lee S., Xu J., Kittler E.L., Zapp M.L., Weng Z. (2008). Endogenous siRNAs derived from transposons and mRNAs in Drosophila somatic cells. Science.

[52] Kawamura Y., Saito K., Kin T., Ono Y., Asai K., Sunohara T., Okada T.N., Siomi M.C., Siomi H. (2008). Drosophila endogenous small RNAs bind to Argonaute 2 in somatic cells. Nature.

[53] Chung W.J., Okamura K., Martin R., Lai E.C. (2008). Endogenous RNA interference provides a somatic defense against Drosophila transposons. Curr. Biol..

[54] Okamura K., Chung W.J., Ruby J.G., Guo H., Bartel D.P., Lai E.C. (2008). The Drosophila hairpin RNA pathway generates endogenous short interfering RNAs. Nature.

[55] Wang X.H., Aliyari R., Li W.X., Li H.W., Kim K., Carthew R., Atkinson P., Ding S.W. (2006). RNA interference directs innate immunity against viruses in adult Drosophila. Science.

[56] Lee Y., Kim M., Han J., Yeom K.H., Lee S., Baek S.H., Kim V.N. (2004). MicroRNA genes are transcribed by RNA polymerase II. EMBO J..

[57] Cai X., Hagedorn C.H., Cullen B.R. (2004). Human microRNAs are processed from capped, polyadenylated transcripts that can also function as mRNAs. RNA.

[58] Borchert G.M., Lanier W., Davidson B.L. (2006). RNA polymerase III transcribes human microRNAs. Nat. Struct. Mol. Biol..

[59] Saini H.K., Griffiths-Jones S., Enright A.J. (2007). Genomic analysis of human microRNA transcripts. Proc. Natl. Acad. Sci. USA.

[60] Finnegan E.F., Pasquinelli A.E. (2013). MicroRNA biogenesis: Regulating the regulators. Crit. Rev. Biochem. Mol. Biol..

[61] Bartel D.P. (2004). MicroRNAs: Genomics, biogenesis, mechanism, and function. Cell.

[62] Denli A.M., Tops B.B., Plasterk R.H., Ketting R.F., Hannon G.J. (2004). Processing of primary microRNAs by the microprocessor complex. Nature.

[63] Gregory R.I., Yan K.P., Amuthan G., Chendrimada T., Doratotaj B., Cooch N., Shiekhattar R. (2004). The microprocessor complex mediates the genesis of microRNAs. Nature.

[64] Landthaler M., Yalcin A., Tuschl T. (2004). The human DiGeorge syndrome critical region gene 8 and its d. Melanogaster homolog are required for miRNA biogenesis. Curr. Biol..

[65] Han J., Lee Y., Yeom K.H., Kim Y.K., Jin H., Kim V.N. (2004). The drosha-DGCR8 complex in primary microRNA processing. Genes Dev..

[66] Lee Y., Ahn C., Han J., Choi H., Kim J., Yim J., Lee J., Provost P., Radmark O., Kim S. (2003). The nuclear RNase III Drosha initiates microRNA processing. Nature.

[67] Bohnsack M.T., Czaplinski K., Gorlich D. (2004). Exportin 5 is a RanGTP-dependent dsRNA-binding protein that mediates nuclear export of pre-miRNAs. RNA.

[68] Lund E., Guttinger S., Calado A., Dahlberg J.E., Kutay U. (2004). Nuclear export of microRNA precursors. Science.

[69] Yi R., Qin Y., Macara I.G., Cullen B.R. (2003). Exportin-5 mediates the nuclear export of pre-microRNAs and short hairpin RNAs. Genes Dev..

[70] Zeng Y., Cullen B.R. (2004). Structural requirements for pre-microRNA binding and nuclear export by exportin 5. Nucleic Acids Res..

[71] Hutvagner G., McLachlan J., Pasquinelli A.E., Balint E., Tuschl T., Zamore P.D. (2001). A cellular function for the RNA-interference enzyme Dicer in the maturation of the let-7 small temporal RNA. Science.

[72] Lee Y.S., Nakahara K., Pham J.W., Kim K., He Z., Sontheimer E.J., Carthew R.W. (2004). Distinct roles for Drosophila Dicer-1 and Dicer-2 in the siRNA/miRNA silencing pathways. Cell.

[73] Forstemann K., Horwich M.D., Wee L., Tomari Y., Zamore P.D. (2007). Drosophila microRNAs are sorted into functionally distinct argonaute complexes after production by dicer-1. Cell.

[74] Tomari Y., Du T., Zamore P.D. (2007). Sorting of Drosophila small silencing RNAs. Cell.

[75] Schwarz D.S., Hutvagner G., Du T., Xu Z., Aronin N., Zamore P.D. (2003). Asymmetry in the assembly of the RNAi enzyme complex. Cell.

[76] Khvorova A., Reynolds A., Jayasena S.D. (2003). Functional siRNAs and miRNAs exhibit strand bias. Cell.

[77] Bartel D.P. (2009). MicroRNAs: Target recognition and regulatory functions. Cell.

[78] He L., Hannon G.J. (2004). MicroRNAs: Small RNAs with a big role in gene regulation. Nat. Rev. Genet..

[79] Ohrt T., Muetze J., Svoboda P., Schwille P. (2012). Intracellular localization and routing of miRNA and RNAi pathway components. Curr. Top. Med. Chem..

[80] Jones-Rhoades M.W., Bartel D.P., Bartel B. (2006). MicroRNAs and their regulatory roles in plants. Annu. Rev. Plant. Biol..

[81] Chendrimada T.P., Gregory R.I., Kumaraswamy E., Norman J., Cooch N., Nishikura K., Shiekhattar R. (2005). TRBP recruits the Dicer complex to Ago2 for microRNA processing and gene silencing. Nature.

[82] Ender C., Meister G. (2010). Argonaute proteins at a glance. J. Cell Sci..

[83] Su H., Trombly M.I., Chen J., Wang X. (2009). Essential and overlapping functions for mammalian Argonautes in microRNA silencing. Genes Dev..

[84] Riley K.J., Yario T.A., Steitz J.A. (2012). Association of Argonaute proteins and microRNAs can occur after cell lysis. RNA.

[85] Meister G., Landthaler M., Patkaniowska A., Dorsett Y., Teng G., Tuschl T. (2004). Human Argonaute2 mediates RNA cleavage targeted by miRNAs and siRNAs. Mol. Cell.

[86] Liu J., Carmell M.A., Rivas F.V., Marsden C.G., Thomson J.M., Song J.J., Hammond S.M., Joshua-Tor L., Hannon G.J. (2004). Argonaute2 is the catalytic engine of mammalian RNAi. Science.

[87] Berezikov E., Chung W.J., Willis J., Cuppen E., Lai E.C. (2007). Mammalian mirtron genes. Mol. Cell.

[88] Ender C., Krek A., Friedlander M.R., Beitzinger M., Weinmann L., Chen W., Pfeffer S., Rajewsky N., Meister G. (2008). A human snoRNA with microRNA-like functions. Mol. Cell.

[89] Babiarz J.E., Ruby J.G., Wang Y., Bartel D.P., Blelloch R. (2008). Mouse es cells express endogenous shRNAs, siRNAs, and other Microprocessor-independent, Dicer-dependent small RNAs. Genes Dev..

[90] Cole C., Sobala A., Lu C., Thatcher S.R., Bowman A., Brown J.W., Green P.J., Barton G.J., Hutvagner G. (2009). Filtering of deep sequencing data reveals the existence of abundant Dicer-dependent small RNAs derived from tRNAs. RNA.

[91] Brameier M., Herwig A., Reinhardt R., Walter L., Gruber J. (2011). Human box C/D snoRNAs with miRNA like functions: Expanding the range of regulatory RNAs. Nucleic Acids Res..

[92] Ono M., Scott M.S., Yamada K., Avolio F., Barton G.J., Lamond A.I. (2011). Identification of human miRNA precursors that resemble box c/d snoRNAs. Nucleic Acids Res..

[93] Scott M.S., Avolio F., Ono M., Lamond A.I., Barton G.J. (2009). Human miRNA precursors with box H/ACA snoRNA features. PLoS Comput. Biol..

[94] Mourelatos Z., Dostie J., Paushkin S., Sharma A., Charroux B., Abel L., Rappsilber J., Mann M., Dreyfuss G. (2002). Mirnps: A novel class of ribonucleoproteins containing numerous microRNAs. Genes Dev..

[95] Farh K.K., Grimson A., Jan C., Lewis B.P., Johnston W.K., Lim L.P., Burge C.B., Bartel D.P. (2005). The widespread impact of mammalian microRNAs on mRNA repression and evolution. Science.

[96] Grimson A., Farh K.K., Johnston W.K., Garrett-Engele P., Lim L.P., Bartel D.P. (2007). MicroRNA targeting specificity in mammals: Determinants beyond seed pairing. Mol. Cell.

[97] Brennecke J., Stark A., Russell R.B., Cohen S.M. (2005). Principles of microRNA-target recognition. PLoS Biol..

[98] Eichhorn S.W., Guo H., McGeary S.E., Rodriguez-Mias R.A., Shin C., Baek D., Hsu S.H., Ghoshal K., Villen J., Bartel D.P. (2014). mRNA destabilization is the dominant effect of mammalian microRNAs by the time substantial repression ensues. Mol. Cell.

[99] Ameres S.L., Zamore P.D. (2013). Diversifying microRNA sequence and function. Nat. Rev. Mol. Cell Biol..

[100] Friedman R.C., Farh K.K., Burge C.B., Bartel D.P. (2009). Most mammalian mRNAs are conserved targets of microRNAs. Genome Res..

[101] Brummelkamp T.R., Bernards R., Agami R. (2002). A system for stable expression of short interfering RNAs in mammalian cells. Science.

[102] Paddison P.J., Caudy A.A., Hannon G.J. (2002). Stable suppression of gene expression by RNAi in mammalian cells. Proc. Natl. Acad. Sci. USA.

[103] Yang S., Tutton S., Pierce E., Yoon K. (2001). Specific double-stranded RNA interference in undifferentiated mouse embryonic stem cells. Mol. Cell. Biol..

[104] Billy E., Brondani V., Zhang H., Muller U., Filipowicz W. (2001). Specific interference with gene expression induced by long, double-stranded RNA in mouse embryonal teratocarcinoma cell lines. Proc. Natl. Acad. Sci. USA.

[105] Zamore P.D., Tuschl T., Sharp P.A., Bartel D.P. (2000). Rnai: Double-stranded RNA directs the ATP-dependent cleavage of mRNA at 21 to 23 nucleotide intervals. Cell.

[106] Martinez J., Patkaniowska A., Urlaub H., Luhrmann R., Tuschl T. (2002). Single-stranded antisense siRNAs guide target RNA cleavage in RNAi. Cell.

[107] Dueck A., Ziegler C., Eichner A., Berezikov E., Meister G. (2012). MicroRNAs associated with the different human Argonaute proteins. Nucleic Acids Res..

[108] Siolas D., Lerner C., Burchard J., Ge W., Linsley P.S., Paddison P.J., Hannon G.J., Cleary M.A. (2005). Synthetic shRNAs as potent RNAi triggers. Nat. Biotechnol..

[109] Isaacs A., Cox R.A., Rotem Z. (1963). Foreign nucleic acids as the stimulus to make interferon. Lancet.

[110] Hardy M.P., Owczarek C.M., Trajanovska S., Liu X., Kola I., Hertzog P.J. (2001). The soluble murine type I interferon receptor Ifnar-2 is present in serum, is independently regulated, and has both agonistic and antagonistic properties. Blood.

[111] Hardy M.P., Sanij E.P., Hertzog P.J., Owczarek C.M. (2003). Characterization and transcriptional analysis of the mouse chromosome 16 cytokine receptor gene cluster. Mamm. Genome.

[112] Hardy M.P., Hertzog P.J., Owczarek C.M. (2002). Multiple regions within the promoter of the murine Ifnar-2 gene confer basal and inducible expression. Biochem. J..

[113] Goubau D., Deddouche S., Reis e Sousa C. (2013). Cytosolic sensing of viruses. Immunity.

[114] Schneider W.M., Chevillotte M.D., Rice C.M. (2014). Interferon-stimulated genes: A complex web of host defenses. Annu. Rev. Immunol..

[115] Schoggins J.W., Rice C.M. (2011). Interferon-stimulated genes and their antiviral effector functions. Curr. Opin. Virol..

[116] Li G., Xiang Y., Sabapathy K., Silverman R.H. (2004). An apoptotic signaling pathway in the interferon antiviral response mediated by RNase l and c-Jun NH2-terminal kinase. J. Biol. Chem..

[117] Ivashkiv L.B., Donlin L.T. (2014). Regulation of type I interferon responses. Nat. Rev. Immunol..

[118] Schlee M., Hartmann G. (2016). Discriminating self from non-self in nucleic acid sensing. Nat. Rev. Immunol..

[119] Levin D., London I.M. (1978). Regulation of protein synthesis: Activation by double-stranded RNA of a protein kinase that phosphorylates eukaryotic initiation factor 2. Proc. Natl. Acad. Sci. USA.

[120] Zilberstein A., Kimchi A., Schmidt A., Revel M. (1978). Isolation of two interferon-induced translational inhibitors: A protein kinase and an oligo-isoadenylate synthetase. Proc. Natl. Acad. Sci. USA.

[121] Hovanessian A.G., Brown R.E., Kerr I.M. (1977). Synthesis of low molecular weight inhibitor of protein synthesis with enzyme from interferon-treated cells. Nature.

[122] Zhou A., Hassel B.A., Silverman R.H. (1993). Expression cloning of 2-5a-dependent RNAase: A uniquely regulated mediator of interferon action. Cell.

[123] Takeuchi O., Akira S. (2010). Pattern recognition receptors and inflammation. Cell.

[124] Luo D., Kohlway A., Pyle A.M. (2013). Duplex RNA activated atpases (dras): Platforms for RNA sensing, signaling and processing. RNA Biol..

[125] Cui S., Eisenacher K., Kirchhofer A., Brzozka K., Lammens A., Lammens K., Fujita T., Conzelmann K.K., Krug A., Hopfner K.P. (2008). The C-terminal regulatory domain is the RNA 5’-triphosphate sensor of RIG-I. Mol. Cell.

[126] Takahasi K., Yoneyama M., Nishihori T., Hirai R., Kumeta H., Narita R., Gale M., Inagaki F., Fujita T. (2008). Nonself RNA-sensing mechanism of RIG-I helicase and activation of antiviral immune responses. Mol. Cell.

[127] Reikine S., Nguyen J.B., Modis Y. (2014). Pattern recognition and signaling mechanisms of RIG-I and MDA5. Front. Immunol..

[128] Wu J., Chen Z.J. (2014). Innate immune sensing and signaling of cytosolic nucleic acids. Annu. Rev. Immunol..

[129] Sohn J., Hur S. (2016). Filament assemblies in foreign nucleic acid sensors. Curr. Opin. Struct. Biol..

[130] Bruns A.M., Leser G.P., Lamb R.A., Horvath C.M. (2014). The innate immune sensor LGP2 activates antiviral signaling by regulating MDA5-RNA interaction and filament assembly. Mol. Cell.

[131] Bruns A.M., Horvath C.M. (2015). LGP2 synergy with MDA5 in rlr-mediated RNA recognition and antiviral signaling. Cytokine.

[132] Venkataraman T., Valdes M., Elsby R., Kakuta S., Caceres G., Saijo S., Iwakura Y., Barber G.N. (2007). Loss of dexd/h box RNA helicase lGP2 manifests disparate antiviral responses. J. Immunol..

[133] Satoh T., Kato H., Kumagai Y., Yoneyama M., Sato S., Matsushita K., Tsujimura T., Fujita T., Akira S., Takeuchi O. (2010). LGP2 is a positive regulator of RIG-I- and MDA5-mediated antiviral responses. Proc. Natl. Acad. Sci. USA.

[134] Rothenfusser S., Goutagny N., DiPerna G., Gong M., Monks B.G., Schoenemeyer A., Yamamoto M., Akira S., Fitzgerald K.A. (2005). The RNA helicase LGP2 inhibits TLR-independent sensing of viral replication by retinoic acid-inducible gene-I. J. Immunol..

[135] Yoneyama M., Kikuchi M., Matsumoto K., Imaizumi T., Miyagishi M., Taira K., Foy E., Loo Y.M., Gale M., Akira S. (2005). Shared and unique functions of the dexd/h-box helicases RIG-I, MDA5, and LGP2 in antiviral innate immunity. J. Immunol..

[136] Si-Tahar M., Blanc F., Furio L., Chopy D., Balloy V., Lafon M., Chignard M., Fiette L., Langa F., Charneau P. (2014). Protective role of LGP2 in influenza virus pathogenesis. J. Infect. Dis..

[137] Chopy D., Pothlichet J., Lafage M., Megret F., Fiette L., Si-Tahar M., Lafon M. (2011). Ambivalent role of the innate immune response in rabies virus pathogenesis. J. Virol..

[138] Suthar M.S., Ramos H.J., Brassil M.M., Netland J., Chappell C.P., Blahnik G., McMillan A., Diamond M.S., Clark E.A., Bevan M.J. (2012). The RIG-I-like receptor LGP2 controls CD8(+) T cell survival and fitness. Immunity.

[139] Sato S., Li K., Kameyama T., Hayashi T., Ishida Y., Murakami S., Watanabe T., Iijima S., Sakurai Y., Watashi K. (2015). The RNA sensor RIG-I dually functions as an innate sensor and direct antiviral factor for hepatitis B virus. Immunity.

[140] Weber M., Sediri H., Felgenhauer U., Binzen I., Banfer S., Jacob R., Brunotte L., Garcia-Sastre A., Schmid-Burgk J.L., Schmidt T. (2015). Influenza virus adaptation PB2-627K modulates nucleocapsid inhibition by the pathogen sensor RIG-I. Cell Host Microbe.

[141] Weissman I.L. (2000). Stem cells: Units of development, units of regeneration, and units in evolution. Cell.

[142] Doetsch F., Caille I., Lim D.A., Garcia-Verdugo J.M., Alvarez-Buylla A. (1999). Subventricular zone astrocytes are neural stem cells in the adult mammalian brain. Cell.

[143] Kuznetsov S.A., Mankani M.H., Gronthos S., Satomura K., Bianco P., Robey P.G. (2001). Circulating skeletal stem cells. J. Cell Biol..

[144] Saiura A., Sata M., Hirata Y., Nagai R., Makuuchi M. (2001). Circulating smooth muscle progenitor cells contribute to atherosclerosis. Nat. Med..

[145] Burke D.C., Graham C.F., Lehman J.M. (1978). Appearance of interferon inducibility and sensitivity during differentiation of murine teratocarcinoma cells in vitro. Cell.

[146] Chen L.L., Yang L., Carmichael G.G. (2010). Molecular basis for an attenuated cytoplasmic dsRNA response in human embryonic stem cells. Cell Cycle.

[147] Witteveldt J., Knol L.I., Macias S. (2019). MicroRNA-deficient mouse embryonic stem cells acquire a functional interferon response. eLife.

[148] Wu X., Dao Thi V.L., Huang Y., Billerbeck E., Saha D., Hoffmann H.H., Wang Y., Silva L.A.V., Sarbanes S., Sun T. (2018). Intrinsic immunity shapes viral resistance of stem cells. Cell.

[149] Bailey C.C., Zhong G., Huang I.C., Farzan M. (2014). IFITM-family proteins: The cell’s first line of antiviral defense. Annu. Rev. Virol..

[150] Wu X., Robotham J.M., Lee E., Dalton S., Kneteman N.M., Gilbert D.M., Tang H. (2012). Productive hepatitis C virus infection of stem cell-derived hepatocytes reveals a critical transition to viral permissiveness during differentiation. PLoS Pathog..

[151] Weber F., Kochs G., Haller O., Staeheli P. (2003). Viral evasion of the interferon system: Old viruses, new tricks. J. Interferon Cytokine Res..

[152] Garcia-Sastre A. (2017). Ten strategies of interferon evasion by viruses. Cell Host Microbe.

[153] Talon J., Horvath C.M., Polley R., Basler C.F., Muster T., Palese P., Garcia-Sastre A. (2000). Activation of interferon regulatory factor 3 is inhibited by the influenza A virus NS1 protein. J. Virol..

[154] Kim M.J., Latham A.G., Krug R.M. (2002). Human influenza viruses activate an interferon-independent transcription of cellular antiviral genes: Outcome with influenza a virus is unique. Proc. Natl. Acad. Sci. USA.

[155] Versteeg G.A., Garcia-Sastre A. (2010). Viral tricks to grid-lock the type I interferon system. Curr. Opin. Microbiol..

[156] Laurent-Rolle M., Morrison J., Rajsbaum R., Macleod J.M.L., Pisanelli G., Pham A., Ayllon J., Miorin L., Martinez C., tenOever B.R. (2014). The interferon signaling antagonist function of yellow fever virus NS5 protein is activated by type I interferon. Cell Host Microbe.

[157] Hammond S.M. (2005). Dicing and slicing: The core machinery of the RNA interference pathway. FEBS Lett..

[158] Weber F., Wagner V., Rasmussen S.B., Hartmann R., Paludan S.R. (2006). Double-stranded RNA is produced by positive-strand RNA viruses and DNA viruses but not in detectable amounts by negative-strand RNA viruses. J. Virol..

[159] Bronkhorst A.W., van Cleef K.W., Vodovar N., Ince I.A., Blanc H., Vlak J.M., Saleh M.C., van Rij R.P. (2012). The DNA virus invertebrate iridescent virus 6 is a target of the Drosophila RNAi machinery. Proc. Natl. Acad. Sci. USA.

[160] Deddouche S., Matt N., Budd A., Mueller S., Kemp C., Galiana-Arnoux D., Dostert C., Antoniewski C., Hoffmann J.A., Imler J.L. (2008). The dexd/h-box helicase dicer-2 mediates the induction of antiviral activity in Drosophila. Nat. Immunol..

[161] Galiana-Arnoux D., Dostert C., Schneemann A., Hoffmann J.A., Imler J.L. (2006). Essential function in vivo for dicer-2 in host defense against RNA viruses in Drosophila. Nat. Immunol..

[162] Sabin L.R., Zheng Q., Thekkat P., Yang J., Hannon G.J., Gregory B.D., Tudor M., Cherry S. (2013). Dicer-2 processes diverse viral RNA species. PLoS ONE.

[163] Marques J.T., Wang J.P., Wang X., de Oliveira K.P., Gao C., Aguiar E.R., Jafari N., Carthew R.W. (2013). Functional specialization of the small interfering RNA pathway in response to virus infection. PLoS Pathog..

[164] Han Y.H., Luo Y.J., Wu Q., Jovel J., Wang X.H., Aliyari R., Han C., Li W.X., Ding S.W. (2011). Rna-based immunity terminates viral infection in adult Drosophila in the absence of viral suppression of RNA interference: Characterization of viral small interfering RNA populations in wild-type and mutant flies. J. Virol..

[165] Mueller S., Gausson V., Vodovar N., Deddouche S., Troxler L., Perot J., Pfeffer S., Hoffmann J.A., Saleh M.C., Imler J.L. (2010). RNAi-mediated immunity provides strong protection against the negative-strand RNA vesicular stomatitis virus in Drosophila. Proc. Natl. Acad. Sci. USA.

[166] van Rij R.P., Saleh M.C., Berry B., Foo C., Houk A., Antoniewski C., Andino R. (2006). The RNA silencing endonuclease Argonaute 2 mediates specific antiviral immunity in Drosophila melanogaster. Genes Dev..

[167] Kemp C., Mueller S., Goto A., Barbier V., Paro S., Bonnay F., Dostert C., Troxler L., Hetru C., Meignin C. (2013). Broad RNA interference-mediated antiviral immunity and virus-specific inducible responses in Drosophila. J. Immunol..

[168] Campbell C.L., Keene K.M., Brackney D.E., Olson K.E., Blair C.D., Wilusz J., Foy B.D. (2008). Aedes aegypti uses RNA interference in defense against sindbis virus infection. BMC Microbiol..

[169] Sanchez-Vargas I., Scott J.C., Poole-Smith B.K., Franz A.W., Barbosa-Solomieu V., Wilusz J., Olson K.E., Blair C.D. (2009). Dengue virus type 2 infections of aedes aegypti are modulated by the mosquito’s RNA interference pathway. PLoS Pathog..

[170] Keene K.M., Foy B.D., Sanchez-Vargas I., Beaty B.J., Blair C.D., Olson K.E. (2004). RNA interference acts as a natural antiviral response to O’nyong-nyong virus (alphavirus; togaviridae) infection of Anopheles gambiae. Proc. Natl. Acad. Sci. USA.

[171] Samuel G.H., Wiley M.R., Badawi A., Adelman Z.N., Myles K.M. (2016). Yellow fever virus capsid protein is a potent suppressor of RNA silencing that binds double-stranded RNA. Proc. Natl. Acad. Sci. USA.

[172] Wilkins C., Dishongh R., Moore S.C., Whitt M.A., Chow M., Machaca K. (2005). RNA interference is an antiviral defence mechanism in caenorhabditis elegans. Nature.

[173] Felix M.A., Ashe A., Piffaretti J., Wu G., Nuez I., Belicard T., Jiang Y., Zhao G., Franz C.J., Goldstein L.D. (2011). Natural and experimental infection of Caenorhabditis nematodes by novel viruses related to nodaviruses. PLoS Biol..

[174] Lu R., Maduro M., Li F., Li H.W., Broitman-Maduro G., Li W.X., Ding S.W. (2005). Animal virus replication and RNAi-mediated antiviral silencing in Caenorhabditis elegans. Nature.

[175] Wassenegger M., Krczal G. (2006). Nomenclature and functions of RNA-directed RNA polymerases. Trends Plant. Sci..

[176] Saleh M.C., Tassetto M., van Rij R.P., Goic B., Gausson V., Berry B., Jacquier C., Antoniewski C., Andino R. (2009). Antiviral immunity in Drosophila requires systemic RNA interference spread. Nature.

[177] Goic B., Vodovar N., Mondotte J.A., Monot C., Frangeul L., Blanc H., Gausson V., Vera-Otarola J., Cristofari G., Saleh M.C. (2013). RNA-mediated interference and reverse transcription control the persistence of RNA viruses in the insect model Drosophila. Nat. Immunol..

[178] Goic B., Stapleford K.A., Frangeul L., Doucet A.J., Gausson V., Blanc H., Schemmel-Jofre N., Cristofari G., Lambrechts L., Vignuzzi M. (2016). Virus-derived DNA drives mosquito vector tolerance to arboviral infection. Nat. Commun..

[179] Tassetto M., Kunitomi M., Andino R. (2017). Circulating immune cells mediate a systemic RNAi-based adaptive antiviral response in Drosophila. Cell.

[180] Gammon D.B., Mello C.C. (2015). RNA interference-mediated antiviral defense in insects. Curr. Opin. Insect Sci..

[181] Nayak A., Tassetto M., Kunitomi M., Andino R. (2013). RNA interference-mediated intrinsic antiviral immunity in invertebrates. Curr. Top. Microbiol. Immunol..

[182] Li H., Li W.X., Ding S.W. (2002). Induction and suppression of RNA silencing by an animal virus. Science.

[183] Chao J.A., Lee J.H., Chapados B.R., Debler E.W., Schneemann A., Williamson J.R. (2005). Dual modes of RNA-silencing suppression by flock house virus protein B2. Nat. Struct. Mol. Biol..

[184] Lingel A., Simon B., Izaurralde E., Sattler M. (2005). The structure of the flock house virus b2 protein, a viral suppressor of RNA interference, shows a novel mode of double-stranded RNA recognition. EMBO Rep..

[185] Singh G., Popli S., Hari Y., Malhotra P., Mukherjee S., Bhatnagar R.K. (2009). Suppression of RNA silencing by Flock house virus B2 protein is mediated through its interaction with the PAZ domain of Dicer. FASEB J..

[186] Aliyari R., Wu Q., Li H.W., Wang X.H., Li F., Green L.D., Han C.S., Li W.X., Ding S.W. (2008). Mechanism of induction and suppression of antiviral immunity directed by virus-derived small RNAs in Drosophila. Cell Host Microbe.

[187] van Cleef K.W., van Mierlo J.T., Miesen P., Overheul G.J., Fros J.J., Schuster S., Marklewitz M., Pijlman G.P., Junglen S., van Rij R.P. (2014). Mosquito and Drosophila entomobirnaviruses suppress dsRNA- and siRNA-induced RNAi. Nucleic Acids Res..

[188] van Mierlo J.T., Bronkhorst A.W., Overheul G.J., Sadanandan S.A., Ekstrom J.O., Heestermans M., Hultmark D., Antoniewski C., van Rij R.P. (2012). Convergent evolution of Argonaute-2 slicer antagonism in two distinct insect RNA viruses. PLoS Pathog..

[189] Nayak A., Berry B., Tassetto M., Kunitomi M., Acevedo A., Deng C., Krutchinsky A., Gross J., Antoniewski C., Andino R. (2010). Cricket paralysis virus antagonizes Argonaute 2 to modulate antiviral defense in Drosophila. Nat. Struct. Mol. Biol..

[190] van Mierlo J.T., Overheul G.J., Obadia B., van Cleef K.W., Webster C.L., Saleh M.C., Obbard D.J., van Rij R.P. (2014). Novel Drosophila viruses encode host-specific suppressors of RNAi. PLoS Pathog..

[191] Obbard D.J., Jiggins F.M., Halligan D.L., Little T.J. (2006). Natural selection drives extremely rapid evolution in antiviral RNAi genes. Curr. Biol..

[192] Svobodova E., Kubikova J., Svoboda P. (2016). Production of small RNAs by mammalian Dicer. Pflugers Arch..

[193] Carmell M.A., Xuan Z., Zhang M.Q., Hannon G.J. (2002). The Argonaute family: Tentacles that reach into RNAi, developmental control, stem cell maintenance, and tumorigenesis. Genes Dev..

[194] Murchison E.P., Partridge J.F., Tam O.H., Cheloufi S., Hannon G.J. (2005). Characterization of Dicer-deficient murine embryonic stem cells. Proc. Natl. Acad. Sci. USA.

[195] Calabrese J.M., Seila A.C., Yeo G.W., Sharp P.A. (2007). RNA sequence analysis defines Dicer’s role in mouse embryonic stem cells. Proc. Natl. Acad. Sci. USA.

[196] Chong M.M., Zhang G., Cheloufi S., Neubert T.A., Hannon G.J., Littman D.R. (2010). Canonical and alternate functions of the microRNA biogenesis machinery. Genes Dev..

[197] Bernstein E., Kim S.Y., Carmell M.A., Murchison E.P., Alcorn H., Li M.Z., Mills A.A., Elledge S.J., Anderson K.V., Hannon G.J. (2003). Dicer is essential for mouse development. Nat. Genet..

[198] Kanellopoulou C., Muljo S.A., Kung A.L., Ganesan S., Drapkin R., Jenuwein T., Livingston D.M., Rajewsky K. (2005). Dicer-deficient mouse embryonic stem cells are defective in differentiation and centromeric silencing. Genes Dev..

[199] Frohn A., Eberl H.C., Stohr J., Glasmacher E., Rudel S., Heissmeyer V., Mann M., Meister G. (2012). Dicer-dependent and -independent Argonaute2 protein interaction networks in mammalian cells. Mol. Cell. Proteom..

[200] Smibert P., Yang J.S., Azzam G., Liu J.L., Lai E.C. (2013). Homeostatic control of Argonaute stability by microRNA availability. Nat. Struct. Mol. Biol..

[201] Bogerd H.P., Whisnant A.W., Kennedy E.M., Flores O., Cullen B.R. (2014). Derivation and characterization of dicer- and microRNA-deficient human cells. RNA.

[202] Backes S., Langlois R.A., Schmid S., Varble A., Shim J.V., Sachs D., tenOever B.R. (2014). The mammalian response to virus infection is independent of small RNA silencing. Cell Rep..

[203] Bogerd H.P., Skalsky R.L., Kennedy E.M., Furuse Y., Whisnant A.W., Flores O., Schultz K.L., Putnam N., Barrows N.J., Sherry B. (2014). Replication of many human viruses is refractory to inhibition by endogenous cellular microRNAs. J. Virol..

[204] Parameswaran P., Sklan E., Wilkins C., Burgon T., Samuel M.A., Lu R., Ansel K.M., Heissmeyer V., Einav S., Jackson W. (2010). Six RNA viruses and forty-one hosts: Viral small RNAs and modulation of small RNA repertoires in vertebrate and invertebrate systems. PLoS Pathog..

[205] Girardi E., Chane-Woon-Ming B., Messmer M., Kaukinen P., Pfeffer S. (2013). Identification of RNase L-dependent, 3’-end-modified, viral small RNAs in Sindbis virus-infected mammalian cells. MBio.

[206] Qiu Y., Xu Y., Zhang Y., Zhou H., Deng Y.Q., Li X.F., Miao M., Zhang Q., Zhong B., Hu Y. (2017). Human virus-derived small RNAs can confer antiviral immunity in mammals. Immunity.

[207] Tsai K., Courtney D.G., Kennedy E.M., Cullen B.R. (2018). Influenza a virus-derived siRNAs increase in the absence of NS1 yet fail to inhibit virus replication. RNA.

[208] Kennedy E.M., Whisnant A.W., Kornepati A.V., Marshall J.B., Bogerd H.P., Cullen B.R. (2015). Production of functional small interfering RNAs by an amino-terminal deletion mutant of human Dicer. Proc. Natl. Acad. Sci. USA.

[209] Donaszi-Ivanov A., Mohorianu I., Dalmay T., Powell P.P. (2013). Small RNA analysis in Sindbis virus infected human HEK293 cells. PLoS ONE.

[210] Xu Y.P., Qiu Y., Zhang B., Chen G., Chen Q., Wang M., Mo F., Xu J., Wu J., Zhang R.R. (2019). Zika virus infection induces RNAi-mediated antiviral immunity in human neural progenitors and brain organoids. Cell Res..

[211] Haasnoot J., de Vries W., Geutjes E.J., Prins M., de Haan P., Berkhout B. (2007). The Ebola virus VP35 protein is a suppressor of RNA silencing. PLoS Pathog..

[212] Cui L., Wang H., Ji Y., Yang J., Xu S., Huang X., Wang Z., Qin L., Tien P., Zhou X. (2015). The nucleocapsid protein of coronaviruses acts as a viral suppressor of RNA silencing in mammalian cells. J. Virol..

[213] Fabozzi G., Nabel C.S., Dolan M.A., Sullivan N.J. (2011). Ebolavirus proteins suppress the effects of small interfering RNA by direct interaction with the mammalian RNA interference pathway. J. Virol..

[214] Garcia-Sastre A., Egorov A., Matassov D., Brandt S., Levy D.E., Durbin J.E., Palese P., Muster T. (1998). Influenza A virus lacking the NS1 gene replicates in interferon-deficient systems. Virology.

[215] Li W.X., Li H., Lu R., Li F., Dus M., Atkinson P., Brydon E.W., Johnson K.L., Garcia-Sastre A., Ball L.A. (2004). Interferon antagonist proteins of influenza and vaccinia viruses are suppressors of RNA silencing. Proc. Natl. Acad. Sci. USA.

[216] Prins K.C., Delpeut S., Leung D.W., Reynard O., Volchkova V.A., Reid S.P., Ramanan P., Cardenas W.B., Amarasinghe G.K., Volchkov V.E. (2010). Mutations abrogating VP35 interaction with double-stranded RNA render ebola virus avirulent in guinea pigs. J. Virol..

[217] Pijlman G.P., Funk A., Kondratieva N., Leung J., Torres S., van der Aa L., Liu W.J., Palmenberg A.C., Shi P.Y., Hall R.A. (2008). A highly structured, nuclease-resistant, noncoding RNA produced by flaviviruses is required for pathogenicity. Cell Host Microbe.

[218] Schnettler E., Sterken M.G., Leung J.Y., Metz S.W., Geertsema C., Goldbach R.W., Vlak J.M., Kohl A., Khromykh A.A., Pijlman G.P. (2012). Noncoding flavivirus RNA displays RNA interference suppressor activity in insect and mammalian cells. J. Virol..

[219] Bidet K., Dadlani D., Garcia-Blanco M.A. (2014). G3BP1, G3BP2 and CAPRIN1 are required for translation of interferon stimulated mRNAs and are targeted by a dengue virus non-coding RNA. PLoS Pathog..

[220] Backes S., Shapiro J.S., Sabin L.R., Pham A.M., Reyes I., Moss B., Cherry S., tenOever B.R. (2012). Degradation of host microRNAs by poxvirus poly(A) polymerase reveals terminal RNA methylation as a protective antiviral mechanism. Cell Host Microbe.

[221] Johnson K.L., Price B.D., Eckerle L.D., Ball L.A. (2004). Nodamura virus nonstructural protein B2 can enhance viral RNA accumulation in both mammalian and insect cells. J. Virol..

[222] Luthra P., Ramanan P., Mire C.E., Weisend C., Tsuda Y., Yen B., Liu G., Leung D.W., Geisbert T.W., Ebihara H. (2013). Mutual antagonism between the Ebola virus VP35 protein and the RIG-I activator pact determines infection outcome. Cell Host Microbe.

[223] Hartman A.L., Towner J.S., Nichol S.T. (2004). A C-terminal basic amino acid motif of Zaire ebolavirus VP35 is essential for type I interferon antagonism and displays high identity with the RNA-binding domain of another interferon antagonist, the NS1 protein of influenza a virus. Virology.

[224] Hu Y., Li W., Gao T., Cui Y., Jin Y., Li P., Ma Q., Liu X., Cao C. (2017). The severe acute respiratory syndrome coronavirus nucleocapsid inhibits type I interferon production by interfering with TRIM25-mediated RIG-I ubiquitination. J. Virol..

[225] Min J.Y., Krug R.M. (2006). The primary function of RNA binding by the influenza A virus NS1 protein in infected cells: Inhibiting the 2’-5’ oligo (a) synthetase/RNAse l pathway. Proc. Natl. Acad. Sci. USA.

[226] Guo Z., Chen L.M., Zeng H., Gomez J.A., Plowden J., Fujita T., Katz J.M., Donis R.O., Sambhara S. (2007). NS1 protein of influenza A virus inhibits the function of intracytoplasmic pathogen sensor, RIG-I. Am. J. Respir. Cell Mol. Biol..

[227] Gack M.U., Albrecht R.A., Urano T., Inn K.S., Huang I.C., Carnero E., Farzan M., Inoue S., Jung J.U., Garcia-Sastre A. (2009). Influenza A virus ns1 targets the ubiquitin ligase TRIM25 to evade recognition by the host viral RNA sensor RIG-I. Cell Host Microbe.

[228] Sullivan C.S., Ganem D. (2005). A virus-encoded inhibitor that blocks RNA interference in mammalian cells. J. Virol..

[229] Bergmann M., Garcia-Sastre A., Carnero E., Pehamberger H., Wolff K., Palese P., Muster T. (2000). Influenza virus NS1 protein counteracts PKR-mediated inhibition of replication. J. Virol..

[230] Benitez A.A., Spanko L.A., Bouhaddou M., Sachs D., tenOever B.R. (2015). Engineered mammalian RNAi can elicit antiviral protection that negates the requirement for the interferon response. Cell Rep..

[231] Kok K.H., Jin D.Y. (2006). Influenza A virus NS1 protein does not suppress RNA interference in mammalian cells. J. Gen. Virol..

[232] Perez J.T., Pham A.M., Lorini M.H., Chua M.A., Steel J., tenOever B.R. (2009). MicroRNA-mediated species-specific attenuation of influenza A virus. Nat. Biotechnol..

[233] Langlois R.A., Albrecht R.A., Kimble B., Sutton T., Shapiro J.S., Finch C., Angel M., Chua M.A., Gonzalez-Reiche A.S., Xu K. (2013). MicroRNA-based strategy to mitigate the risk of gain-of-function influenza studies. Nat. Biotechnol..

[234] Langlois R.A., Varble A., Chua M.A., Garcia-Sastre A., tenOever B.R. (2012). Hematopoietic-specific targeting of influenza A virus reveals replication requirements for induction of antiviral immune responses. Proc. Natl. Acad. Sci. USA.

[235] Varble A., Chua M.A., Perez J.T., Manicassamy B., Garcia-Sastre A., tenOever B.R. (2010). Engineered RNA viral synthesis of microRNAs. Proc. Natl. Acad. Sci. USA.

[236] Pare J.M., Sullivan C.S. (2014). Distinct antiviral responses in pluripotent versus differentiated cells. PLoS Pathog..

[237] Tam O.H., Aravin A.A., Stein P., Girard A., Murchison E.P., Cheloufi S., Hodges E., Anger M., Sachidanandam R., Schultz R.M. (2008). Pseudogene-derived small interfering RNAs regulate gene expression in mouse oocytes. Nature.

[238] Hertzog P.J., Hwang S.Y., Kola I. (1994). Role of interferons in the regulation of cell proliferation, differentiation, and development. Mol. Reprod. Dev..

[239] Qi J., Yu J.Y., Shcherbata H.R., Mathieu J., Wang A.J., Seal S., Zhou W., Stadler B.M., Bourgin D., Wang L. (2009). MicroRNAs regulate human embryonic stem cell division. Cell Cycle.

[240] Garcia-Perez J.L., Widmann T.J., Adams I.R. (2016). The impact of transposable elements on mammalian development. Development.

[241] Ma E., MacRae I.J., Kirsch J.F., Doudna J.A. (2008). Autoinhibition of human Dicer by its internal helicase domain. J. Mol. Biol..

[242] Provost P., Dishart D., Doucet J., Frendewey D., Samuelsson B., Radmark O. (2002). Ribonuclease activity and RNA binding of recombinant human Dicer. EMBO J..

[243] Flemr M., Malik R., Franke V., Nejepinska J., Sedlacek R., Vlahovicek K., Svoboda P. (2013). A retrotransposon-driven dicer isoform directs endogenous small interfering RNA production in mouse oocytes. Cell.

[244] Peaston A.E., Evsikov A.V., Graber J.H., de Vries W.N., Holbrook A.E., Solter D., Knowles B.B. (2004). Retrotransposons regulate host genes in mouse oocytes and preimplantation embryos. Dev. Cell.

[245] Li S., Wang L., Berman M., Kong Y.Y., Dorf M.E. (2011). Mapping a dynamic innate immunity protein interaction network regulating type I interferon production. Immunity.

[246] Takahashi T., Nakano Y., Onomoto K., Murakami F., Komori C., Suzuki Y., Yoneyama M., Ui-Tei K. (2018). LGP2 virus sensor regulates gene expression network mediated by trbp-bound microRNAs. Nucleic Acids Res..

[247] Seo G.J., Kincaid R.P., Phanaksri T., Burke J.M., Pare J.M., Cox J.E., Hsiang T.Y., Krug R.M., Sullivan C.S. (2013). Reciprocal inhibition between intracellular antiviral signaling and the RNAi machinery in mammalian cells. Cell Host Microbe.

[248] Cloonan N., Brown M.K., Steptoe A.L., Wani S., Chan W.L., Forrest A.R., Kolle G., Gabrielli B., Grimmond S.M. (2008). The miR-17-5p microRNA is a key regulator of the G1/S phase cell cycle transition. Genome Biol..

[249] Gregersen L.H., Jacobsen A.B., Frankel L.B., Wen J., Krogh A., Lund A.H. (2010). MicroRNA-145 targets YES and STAT1 in colon cancer cells. PLoS ONE.

[250] Lee T.Y., Ezelle H.J., Venkataraman T., Lapidus R.G., Scheibner K.A., Hassel B.A. (2013). Regulation of human RNase-L by the miR-29 family reveals a novel oncogenic role in chronic myelogenous leukemia. J. Interferon Cytokine Res..

[251] Ostermann E., Tuddenham L., Macquin C., Alsaleh G., Schreiber-Becker J., Tanguy M., Bahram S., Pfeffer S., Georgel P. (2012). Deregulation of type I IFN-dependent genes correlates with increased susceptibility to cytomegalovirus acute infection of Dicer mutant mice. PLoS ONE.

